# Cross-and-Capture: A Dual-Ligand PAMAM Nanocarrier for Sequential BBB Penetration and Ischemic Lesion Retention of Breviscapine

**DOI:** 10.3390/molecules31193363

**Published:** 2026-09-22

**Authors:** Haoran Guo, Dandan Han, Xin Liu

**Affiliations:** School of Materials Science and Chemical Engineering, Harbin University of Science and Technology, Harbin 150040, China; 19984187566@163.com (H.G.); 18845613098@163.com (D.H.)

**Keywords:** breviscapine, blood–brain barrier, drug delivery system, dual-targeting, ischemic stroke, PAMAM dendrimer

## Abstract

The clinical management of ischemic stroke is severely constrained by the blood–brain barrier (BBB), which restricts therapeutic drug access to the brain parenchyma, and the rapid systemic clearance of neuroprotective agents such as breviscapine (Bre). To overcome this dual challenge, we engineered a dual-targeting nanocarrier, L57&PGP-PEG-PAMAM, designed to sequentially traverse the BBB and anchor within the ischemic inflammatory microenvironment. This construct employs a PEGylated fifth-generation polyamidoamine (PAMAM) dendrimer scaffold functionalized with two distinct peptides: L57, which mediates transcytosis via LRP1 receptors on the BBB, and PGP, which targets CXCR2 receptors overexpressed on infiltrating neutrophils at the lesion. The resulting nano particles exhibited a hydrodynamic diameter of 140.2 ± 0.6 nm (zeta potential: 9.32 mV) and encapsulated Bre with a loading capacity of 20.12% and encapsulation efficiency of 71.78%, displaying sustained release (54.6% over 72 h). Crucially, in vivo fluorescence imaging confirmed markedly superior brain accumulation relative to non-targeted and PEGylated-only controls, while in a rat MCAO model, the formulation reduced infarct volume to 21.7 ± 6.4%, improved neurological scores to 1.52 ± 0.28, and suppressed pro-inflammatory IL-15/IL-27 levels—collectively outperforming free Bre. This work establishes a robust “cross-and-capture” strategy that effectively decouples BBB penetration from lesion-specific retention for ischemic stroke therapy.

## 1. Introduction

Effective pharmacotherapy for ischemic stroke is limited not by the absence of neuroprotective candidates but by the inability to deliver them to the ischemic brain in sustained [1], therapeutically relevant concentrations. The blood–brain barrier (BBB), formed by tight-junction-sealed cerebral endothelium, excludes the vast majority of systemically administered agents from the brain parenchyma [2], while the short plasma half-life and poor aqueous solubility of agents such as breviscapine (Bre)—a flavonoid preparation from *Erigeron breviscapus* with established vasodilatory, antiplatelet [3], and anti-ischemic-reperfusion activities—further prevent meaningful drug accumulation at the infarct site. The central delivery challenge in this context is therefore twofold: first, crossing the endothelial barrier, second, retaining the payload at the evolving inflammatory lesion rather than allowing it to redistribute diffusely through the brain parenchyma [4,5,6].

Individual technological elements capable of addressing each of these barriers have been validated independently. PAMAM dendrimers, with their monodisperse hyperbranched architecture and multivalent surface chemistry, provide well-defined internal cavities for drug encapsulation and permit sequential [7], orthogonal surface functionalization. PEGylation of high-generation PAMAM masks the cationic periphery responsible for cytotoxicity while extending systemic circulation. The synthetic peptide L57 engages LRP1 on brain capillary endothelium to mediate receptor-driven transcytosis across the intact BBB, and the collagen-mimetic tripeptide PGP targets CXCR2 [8,9,10,11,12], upregulated on neutrophils infiltrating the ischemic penumbra, to provide inflammatory-site anchoring. However, each of these elements has been implemented in isolation. Single-ligand carriers bearing only a BBB-shuttling moiety traverse the endothelium but lack any mechanism to concentrate drug at the ischemic focus [13], whereas inflammation-homing ligands such as PGP cannot engage their receptors unless the carrier has already crossed the BBB. No existing PAMAM-based formulation integrates both functionalities in a sequential architecture within a single nanoparticle, leaving the two delivery barriers—endothelial exclusion and parenchymal dilution—independently addressed but never jointly resolved [14,15,16,17,18].

To close this specific gap, the present study introduces L57&PGP-PEG-PAMAM, a fifth-generation PAMAM dendrimer engineered with a sequential “cross-and-capture” dual-targeting architecture. The design addresses each identified limitation through three integrated structural elements [19,20]. PEGylation via NHS-PEG-MAL grafting constitutes the foundational modification: by neutralizing the dense cationic surface and generating a hydrophilic stealth corona, it attenuates the membrane-disruptive cytotoxicity inherent to unmodified high-generation PAMAM while extending circulatory half-life sufficiently to permit productive engagement with the BBB [21]. The LRP1-binding peptide L57, conjugated to the PEG (Figure 1) terminus via maleimide–thiol chemistry, provides the first functional module—active, receptor-mediated transcytosis across brain capillary endothelium—replacing reliance on passive diffusion and directly overcoming the endothelial permeability barrier [22,23,24,25]. PGP, co-conjugated as a secondary homing ligand, provides the second functional module: once the carrier has traversed the BBB, PGP engages CXCR2 on infiltrating neutrophils to anchor the nanoparticle within the ischemic inflammatory microenvironment, preventing post-transcytosis redistribution and concentrating the payload at the lesion. Bre is physically encapsulated within the dendrimer interior [26], and the PEG–peptide corona imposes an additional diffusion barrier on drug efflux, generating a sustained release profile suited to maintaining local therapeutic concentrations over a clinically relevant timeframe [27,28,29,30]. This architecture thus decouples barrier penetration from lesion-specific retention within a single nanoparticle, converting two independently validated targeting strategies into a unified, sequential delivery mechanism [31,32].

Therefore, the aim of this study is to construct and validate this dual-ligand PAMAM nanocarrier as a platform that enhances the brain bioavailability and neuroprotective efficacy of breviscapine in ischemic stroke. The following sections describe the stepwise synthesis and physicochemical characterization of L57&PGP-PEG-PAMAM, in vivo brain-targeting imaging and pharmacodynamic evaluation in a rat middle cerebral artery occlusion model, and the translational implications of the sequential dual-targeting design. Looking forward, the modular conjugation chemistry employed here permits straightforward substitution or addition of targeting ligands, suggesting that this dendrimer scaffold could be adapted to other neuroinflammatory conditions in which receptor-mediated barrier traversal and immune-cell anchoring are simultaneously required.

## 2. Materials and Methods

### 2.1. Reagents and Materials

NHS-PEG-MAL (MW = 2000 Da) was purchased from Shanghai Fanshuo Biotechnology Co., Ltd. (Shanghai, China). Anhydrous methanol (analytical grade) was obtained from J&K Scientific Ltd. (Beijing, China). Ethylenediamine (analytical grade) was supplied by Shanghai Macklin Biochemical Co., Ltd. (Shanghai, China). Methyl acrylate (analytical grade) and anhydrous diethyl ether (analytical grade) were purchased from Shanghai Aladdin Bio-Chem Technology Co., Ltd. (Shanghai, China). Thin-layer chromatography (TLC) silica gel GF254 was obtained from Qingdao Jiyida Silica Gel Reagent Co., Ltd. (Qingdao, China). Breviscapine (analytical grade, scutellarin content ≥ 85%) was purchased from Nanjing Chunqiu Biological Engineering Co., Ltd. (Nanjing, China) (see Supplementary Materials). L57 (sequence: TWPKHFDKHTFYSILKLGKH-Cys, C-terminal cysteine for maleimide coupling, MW ≈ 2553 Da) and PGP (sequence: Cys-Pro-Gly-Pro, N-terminal cysteine for maleimide coupling, MW ≈ 460 Da) were custom-synthesized by Shanghai Fanshuo Biotechnology Co., Ltd. (Shanghai, China). Carboxymethylcellulose sodium (CMC-Na, analytical grade) was obtained from Shanghai Aladdin Bio-Chem Technology Co., Ltd. (Shanghai, China). All reagents were used as received without further purification.

### 2.2. Instrumentation

^1^H and ^13^C nuclear magnetic resonance (NMR) spectra were recorded on a Bruker Avance III HD 500 MHz spectrometer (Bruker, Fällanden, Switzerland). Fourier-transform infrared (FTIR) spectra were acquired on a Nicolet 60SXB FTIR spectrometer (Thermo Fisher Scientific, Waltham, MA, USA). Morphological characterization was performed using a JSM-5610LV scanning electron microscope (JEOL, Tokyo, Japan) and a SUPRA 55 field emission scanning electron microscope (Carl Zeiss, Jena, Germany). Particle size distribution and zeta potential measurements were conducted on a BT-90 nano laser particle size analyzer (Dandong Baite Instrument Co., Ltd., Dandong, China). Solution pH was measured using a WIGGENS pH610 pH meter (WIGGENS, Wuppertal, Germany). Mass determination was performed on a Sartorius SECURA213-1CN analytical balance (Sartorius AG, Göttingen, Germany). Solvent removal was carried out on an RE-52A rotary evaporator (Shanghai Yarong Biochemical Instrument Factory, Shanghai, China). Drying operations were conducted using a ZK072 vacuum drying oven (Shanghai Experimental Instrument Co., Ltd., Shanghai, China) and a SCIENTZ-12N vacuum freeze dryer (Ningbo Scientz Biotechnology Co., Ltd., Ningbo, China). Centrifugation was performed on a TG16-WS benchtop high-speed centrifuge (Xiangyi Instrument Development Co., Ltd., Changsha, China). Vacuum was provided by a 2XZ-4 rotary vane vacuum pump (Shanghai Lichen Technology Co., Ltd., Shanghai, China). Magnetic stirring was carried out using a CL-200 magnetic stirrer (Gongyi Yuhua Instrument Co., Ltd., Gongyi City, China) (see Supplementary Materials).

### 2.3. Animals

Sprague-Dawley (SD) rats (male, 200–220 g) and imprinting control region (ICR) mice (male, 20–25 g) were provided by the Shanghai Slac Laboratory Animal Co., Ltd. (Shanghai, China). All animals were housed at least 1 week to adapt to the new environment with a temperature of 22 + 1 °C and a relative humidity of 65% + 5% under a 12 h light/dark cycle. Animal experiments were approved by the Institutional Animal Care and Use Committee of Harbin University of Science and Technology under the approved in accordance with the guidelines followed for the welfare and ethics of the laboratory animals of Harbin University of Science and Technology (HRBUST-EC-2023-0228).

### 2.4. Synthesis of G5.0 PAMAM Dendrimers

PAMAM dendrimers were synthesized via the divergent method [33,34]. G0.5 PAMAM was prepared by Michael addition: EDA was dissolved in methanol under nitrogen protection, and an 8-fold molar excess of MA was added dropwise (1 drop/s) via a pressure-equalizing funnel in an ice bath. The reaction proceeded in the dark under nitrogen for 24 h. Excess MA and methanol were removed by rotary evaporation at 50 °C, and the crude product was washed repeatedly with anhydrous methanol followed by freeze-drying. Full-generation PAMAM (G1.0) was obtained by amidation: G0.5 PAMAM was dissolved in methanol, and a 24-fold molar excess of EDA was added dropwise under identical conditions. The product was purified by rotary evaporation, methanol washing, diethyl ether extraction, and freeze-drying. Alternating half-generation (MA addition) and full-generation (EDA amidation) steps were repeated iteratively to yield G5.0 PAMAM. Key ^1^H-NMR assignments (D_2_O, 500 MHz): δ2.30 (t, –CH_2_CONH–, position c), 2.47 (t, –NCH_2_CH_2_CO–, position a), 2.58 (t, –NCH_2_CH_2_NH–, position b), 2.69 (t, –CONHCH_2_CH_2_N–, position f), 2.84 (t, –NHCH_2_CH_2_CONH–, position g), 3.10 (t, –NHCH_2_CH_2_NH–, position e), and 3.21 ppm (t, core –NCH_2_CH_2_–, position d).

### 2.5. Synthesis of MAL-PEG-PAMAM

G5.0 PAMAM (25 mg, 0.87 µmol; theoretical MW ≈ 28,826 Da, 128 surface primary amines per dendrimer) was dissolved in 5 mL of PBS buffer (pH 8.36) under nitrogen protection with stirring. NHS-PEG-MAL (55.52 mg, 27.76 µmol; 32 molar equivalents relative to PAMAM, corresponding to a theoretical modification of 25% of surface primary amines, i.e., 32 out of 128 amines) was separately dissolved in 5 mL of the same buffer by sonication. The PEG solution was added dropwise to the PAMAM solution, and the mixture was stirred in the dark under nitrogen for 24 h. The product was purified by repeated ultrafiltration (MWCO = 10 kDa), and complete removal of unreacted PEG was confirmed by thin-layer chromatography (TLC; developing solvent: dichloromethane/methanol = 5:3, visualized by iodine vapor). The purified MAL-PEG-PAMAM was freeze-dried.

^1^H-NMR analysis (D_2_O, 500 MHz) of PAMAM-PEG: PAMAM backbone signals were retained at δ 2.34 (position c), 2.56 (position a), 2.74 (position b), 2.84 (position g), 3.03 (position f), 3.21 (position d), and 3.40 ppm (position e); the PEG repeating unit -(OCH_2_CH_2_)n- appeared at δ 3.62 ppm; methylene protons adjacent to the newly formed amide linkage (-NHCOCH_2_CH_2_O-) appeared at δ 3.86 ppm. From integration of the PEG resonance at 3.62 ppm (approximately 180 H per PEG_2000_chain, corresponding to ~45 -OCH_2_CH_2_- repeat units) relative to the PAMAM core methylene signal at 3.21 ppm (4 H, set to 1 for normalization), the grafted PEG-to-PAMAM ratio was determined to be approximately 28:1, indicating that roughly 22% of the 128 surface primary amines were successfully modified—slightly below the theoretical 25% target, consistent with incomplete NHS-ester conversion under aqueous conditions. Freeze-drying was performed using a SCIENTZ-12N lyophilizer under the following controlled conditions: samples were pre-frozen at −40 °C for 4 h, then subjected to primary drying at −20 °C under 10 Pa vacuum for 24 h, followed by secondary drying at 25 °C under 5 Pa vacuum for 6 h. These parameters were selected to minimize thermal stress on the PEG–amide linkages and to preserve the hydrodynamic integrity of the dendrimer–PEG conjugate.

### 2.6. Synthesis of L57&PGP-PEG-PAMAM

MAL-PEG-PAMAM (10 mg, ~0.29 µmol based on ~28 PEG chains per dendrimer) was dissolved in 2 mL of PBS buffer (pH 7.0) under nitrogen. L57 (2.21 mg, 0.87 µmol; 3.0 molar equivalents relative to total available maleimide sites) and PGP (0.32 mg, 0.70 µmol; 2.4 molar equivalents relative to total available maleimide sites) were separately dissolved in 2 mL of PBS (pH 7.0) by sonication and combined with the dendrimer solution. The reaction mixture was stirred in the dark under nitrogen for 24 h, during which the terminal thiol groups of the peptides underwent Michael addition with the maleimide moieties on the PEG spacer. The L57:PGP molar feed ratio was set at approximately 1.2:1. This ratio was selected on the basis of the following rationale: L57 must be present in slight excess to ensure sufficient LRP1 engagement for BBB transcytosis (the rate-limiting first step), while PGP requires fewer copies per particle because CXCR2 on neutrophils is densely expressed at the ischemic site and multivalent binding is not necessary for effective anchoring. The product was purified by ultrafiltration (MWCO = 10 kDa) and freeze-dried to yield L57&PGP-PEG-PAMAM.

^1^H-NMR confirmation (D_2_O, 500 MHz): aromatic proton signals attributable to PGP appeared at δ 6.80, 7.22, and 7.36 ppm; aliphatic methyl/methylene resonances attributable to L57 appeared at δ0.85–1.15 ppm, consistent with covalent peptide conjugation.

To assess batch reproducibility, three independent dendrimer-peptide conjugation batches were prepared under identical conditions. ^1^H-NMR spectra of all three batches showed consistent PGP aromatic signals (δ6.80–7.36) and L57 aliphatic signals (δ0.85–1.15) with comparable relative integrations, and DLS measurements yielded hydrodynamic diameters of 139.4 ± 0.8, 140.2 ± 0.6, and 141.0 ± 0.9 nm, indicating that the conjugation procedure is reproducible and that the final formulation used in subsequent in vitro and in vivo studies was not an isolated successful batch.

### 2.7. Preparation of Breviscapine-Loaded Nanocarriers

Each carrier material (PAMAM, PAMAM-PEG, or L57&PGP-PEG-PAMAM; 5 mg) was dissolved in 5 mL of deionized water under stirring. Breviscapine powder was then added incrementally in small portions to the aqueous dendrimer solution with continuous stirring; the drug dissolved progressively into the solution until no further dissolution was observed visually. The mixture was stirred in the dark for an additional 8 h. Unencapsulated drug was removed by filtration through a 0.22 µm syringe filter. The filtrate was freeze-dried to obtain PAMAM/Bre, PAMAM-PEG/Bre, and L57&PGP-PEG-PAMAM/Bre as pale yellow powders.

### 2.8. Characterization

Chemical structures were verified by Fourier-transform infrared spectroscopy (FT-IR; Nicolet T60SXB, Nicolet Instrument Corporation, Madison, WI, USA) and proton nuclear magnetic resonance spectroscopy (^1^H-NMR; Bruker AVANCE III HD, 500 MHz, Bruker, Rheinstetten, Germany). Nanoparticle morphology was examined by scanning electron microscopy (SEM; JEOL JSM-5610LV, JEOL Ltd., Tokyo, Japan) and transmission electron microscopy (TEM; Zeiss SUPRA 55, Carl Zeiss, Oberkochen, Germany). Hydrodynamic diameter and zeta potential were determined by dynamic light scattering (DLS) using a BT-90 nano laser particle size analyzer (Dandong Bettersize Instruments Ltd., Dandong, China).

### 2.9. HPLC Method Development and Validation

The detection wavelength for Bre was determined by UV-visible spectrophotometry, with the maximum absorption at 335 nm. HPLC analysis was performed on a Thermo Scientific UltiMate 3000 system equipped with a C18 column (250 mm × 4.6 mm, 5 µm). The mobile phase consisted of water/acetonitrile (80:20, *v*/*v*) at a flow rate of 1.0 mL/min; column temperature was maintained at 30 °C; injection volume was 20 µL; detection wavelength was 335 nm. The retention time of Bre was 9.954 min. A calibration curve was constructed over the concentration range of 1–100 µg/mL, yielding the linear regression equation A = 12.37C + 0.36 (R^2^ = 0.9998). Method specificity, precision (RSD = 0.408%, *n* = 6), and recovery (mean recovery: 99.81–100.31%) were validated.

### 2.10. Drug Loading, Encapsulation Efficiency, and In Vitro Release

Drug loading capacity (DL) and encapsulation efficiency (EE) were determined by dissolving L57&PGP-PEG-PAMAM/Bre (5 mg) in chromatographic-grade methanol, followed by sonication and centrifugation (6000 rpm, 10 min). The supernatant was analyzed by HPLC in triplicate. In vitro release was evaluated using the dialysis method: each formulation (equivalent Bre mass) and free Bre were loaded into dialysis bags (MWCO = 3500 Da) and immersed in PBS (pH 7.4) at 37 ± 1 °C with shaking at 100 rpm. At predetermined time points (0, 0.25, 0.5, 1, 2, 4, 8, 12, 24, 48, and 72 h), 1 mL aliquots were withdrawn and replaced with fresh medium. Released Bre was quantified by HPLC in triplicate.

### 2.11. In Vivo Brain-Targeting Evaluation

DiR-labeled nanocarriers were prepared by adding 100 µL of DiR/DMSO solution (1 mg/mL) to each carrier at a molar ratio of 1:10, followed by addition of 5 mL PBS (pH 7.4) and stirring for 12 h. Healthy ICR mice (22 g; *n* = 2 per group) were anesthetized with 4% chloral hydrate (intraperitoneal injection) and administered DiR-labeled PAMAM, PAMAM-PEG, or L57&PGP-PEG-PAMAM via tail vein injection. Whole-body fluorescence images were acquired at 1, 3, and 5 h post-injection using a Bruker IS4000MM (Fällanden, Switzerland) in vivo imaging system. At 5 h, mice were sacrificed, and major organs (brain, heart, liver, spleen, lung, and kidney) were excised for ex vivo fluorescence imaging.

### 2.12. In Vivo Pharmacodynamic Study

#### 2.12.1. MCAO Model Establishment

Male SD rats (200–220 g; Shanghai SLAC Laboratory Animal Co., Ltd., Shanghai, China) were fasted for 12 h prior to surgery and anesthetized with chloral hydrate. A midline cervical incision was made to expose the bilateral carotid arteries. The external carotid artery was ligated, and loose ligatures were placed around the internal carotid artery and left common carotid artery. A small incision was made approximately 3 mm from the carotid bifurcation, and a 0.28 mm nylon monofilament suture was advanced approximately 1.9 cm into the internal carotid artery to occlude the middle cerebral artery. After 2 h of occlusion, the suture was withdrawn to permit reperfusion. Sham-operated rats underwent identical procedures without suture insertion.

#### 2.12.2. Experimental Groups and Treatment

Forty male SD rats were randomly allocated into four groups (*n* = 10): (A) Sham group: surgery without occlusion, daily saline (2 mL/kg); (B) MCAO group: occlusion/reperfusion, daily saline (2 mL/kg); (C) Bre group: occlusion/reperfusion, daily 0.5% CMC-Na Bre solution (10 mL/kg); (D) L57&PGP-PEG-PAMAM/Bre group: occlusion/reperfusion, daily L57&PGP-PEG-PAMAM/Bre dispersed in 0.5% CMC-Na aqueous solution (10 mL/kg). 0.5% CMC-Na was used as a suspending vehicle for both the free drug and the nanocarrier formulation to ensure uniform dosing and prevent sedimentation during oral gavage administration. All treatments were administered once daily by oral gavage for 7 consecutive days.

#### 2.12.3. Neurological Function Scoring

Neurological deficits were assessed 24 h after reperfusion using a 5-point scoring system: 0 = normal limb movement; 1 = contralateral forelimb flexion; 2 = involuntary circling toward the contralateral side during walking; 3 = severe lateral circling; 4 = absence of spontaneous movement with loss of consciousness.

#### 2.12.4. Infarct Volume Measurement

Rats were sacrificed and brains were removed intact. Coronal sections (approximately 2 mm thick) were stained with 2,3,5-triphenyltetrazolium chloride (TTC) at 37 °C, fixed with formalin, and photographed. Infarct volume was expressed as a percentage of total brain volume.

#### 2.12.5. Histopathological Examination

Brains were fixed in 4% formalin for 12 h, dehydrated, embedded in paraffin, sectioned at 4 µm thickness, and stained with hematoxylin and eosin (H&E). Sections were examined under a Nikon ECLIPSE Ni upright microscope.

#### 2.12.6. Inflammatory Cytokine Measurement

Brain tissues were harvested 24 h post-reperfusion under cold conditions, blotted to remove blood, and weighed. Right hemisphere tissues were homogenized in cold saline using a high-speed homogenizer, centrifuged, and supernatants were collected. IL-15 and IL-27 concentrations were determined by sandwich ELISA (R&D Systems, Minneapolis, MN, USA) according to the manufacturer’s instructions.

### 2.13. Statistical Analysis

Data are expressed as mean ± standard deviation. Statistical comparisons were performed by one-way analysis of variance (ANOVA) using SPSS 19.0 software. *p* < 0.05 or *p* < 0.01 was considered statistically significant.

## 3. Results and Discussion

### 3.1. Synthesis and Structural Characterization

The successful synthesis of each intermediate was confirmed by FT-IR and ^1^H-NMR spectroscopy. The FT-IR spectrum of G5.0 PAMAM (Figure 2) displayed characteristic absorptions at 3290 and 3084 cm^−1^(N–H stretching of terminal primary amines), 2939 and 2854 cm^−1^ (C-H stretching of methylene groups), 1647 cm^−1^ (C=O stretching of amide bonds), 1557 cm^−1^ (N-H bending of amide bonds), 1441 cm^−1^ (C–H bending), and 1204 cm^−1^ (C-N symmetric stretching), consistent with the expected PAMAM structure. Following PEGylation, the amine absorption bands at 3290 and 3084 cm^−1^ were markedly attenuated and merged into a broad single peak, while a new cluster of characteristic peaks appeared in the 1400–1100 cm^−1^ region attributable to NHS-PEG-MAL, confirming successful PEG conjugation.

Following structural confirmation, purification efficacy was assessed by TLC. As shown in Figure 3, the PAMAM-PEG product (lane 1) exhibited a single spot at the baseline with no detectable free PEG migration (lane 2 shows the PEG reference migrating to Rf ≈ 0.6), confirming complete removal of unreacted PEG after ultrafiltration. Notably, this dual-indicator spectroscopic confirmation—loss of terminal amine signal coupled with gain of PEG-specific ether vibrations—provides a more stringent structural verification than the single-parameter amine attenuation criterion previously employed for dendrimer surface functionalization [10], thereby strengthening the assignment of complete PEGylation in the present system (see Supplementary Materials).

^1^H-NMR spectra provided complementary structural evidence. The G5.0 PAMAM spectrum (Figure 4) exhibited characteristic resonances at 2.30, 2.47, 2.58, 2.69, 2.84, 3.10, and 3.21 ppm, assignable to the methylene protons at positions c, a, b, f, g, e, and d of the dendrimer backbone, respectively. The peak at 2.84 ppm (position g), located between the signals at positions d (3.21 ppm) and e (3.10 ppm) in chemical shift order, corresponds to the –NHCH_2_CH_2_CONH– methylene protons in the interior branching units; this signal was included in the integration and is consistent with the expected G5 structure. In the PAMAM-PEG spectrum (Figure 5), these PAMAM signals were retained at 2.34, 2.56, 2.74, 2.84, 3.03, 3.21, and 3.40 ppm, while a new prominent peak at 3.62 ppm corresponding to the repeating –CH_2_CH_2_O– units of PEG and a signal at 3.86 ppm attributed to the methylene protons adjacent to the amide linkage confirmed successful PEGylation. Integration of the PEG signal at 3.62 ppm against the PAMAM core methylene signal at 3.21 ppm (normalized to 1) yielded a grafted PEG-to-PAMAM ratio of approximately 28:1, corresponding to modification of ~22% of the 128 surface primary amines. The ^1^H-NMR spectrum of L57&PGP-PEG-PAMAM (Figure 6) revealed weak but identifiable signals in the aromatic region (6.80, 7.22 and 7.36 ppm) attributable to PGP and in the aliphatic region (0.85–1.15 ppm) attributable to L57, confirming covalent attachment of both targeting peptides. Semi-quantitative comparison of the peptide signal integrations with the PEG backbone signal indicated that approximately 70–80% of the available maleimide sites were consumed by peptide conjugation, consistent with near-complete conversion expected for thiol–maleimide chemistry under these conditions.

### 3.2. Morphology and Particle Size

SEM imaging (Figure 7A,B) revealed that L57&PGP-PEG-PAMAM nanoparticles exhibited an ellipsoidal morphology with relatively uniform size distribution and good inter-particle dispersity. Localized clustering observed in some fields is consistent with the known tendency of PEGylated dendrimers to form reversible aggregates upon dehydration during SEM sample preparation, as documented for similar PAMAM-PEG systems [33,34]; this artifact does not reflect the dispersion state in aqueous suspension, where DLS confirms monomodal size distribution.

TEM (Figure 8A,B) confirmed a quasi-spherical morphology. Multiple representative particles are visible across both panels, with individual particle diameters ranging from 135 to 145 nm, consistent with the ensemble-average hydrodynamic size measured by DLS. The agreement between TEM (dry-state geometric diameter) and DLS (hydrated hydrodynamic diameter) supports the structural integrity of the nanoparticles in solution.

DLS measurements (Figure 9) yielded average hydrodynamic diameters of 58.9 ± 0.5 nm (PDI: 0.12 ± 0.01) for G5.0 PAMAM, 133.6 ± 0.4 nm (PDI: 0.15 ± 0.02) for PAMAM-PEG, and 140.2 ± 0.6 nm (PDI: 0.18 ± 0.02) for L57&PGP-PEG-PAMAM. The progressive size increase reflects sequential surface modification: PEG chain grafting substantially enlarged the hydrodynamic shell, while peptide conjugation contributed a modest additional increment. The PDI values below 0.2 for all formulations indicate narrow, monomodal size distributions suitable for reproducible biological evaluation. Zeta potential decreased from +30.0 mV (PAMAM) to +3.74 mV (PAMAM-PEG), confirming effective charge shielding by the neutral PEG corona. The slight increase to +9.32 mV upon peptide conjugation is consistent with the introduction of positively charged peptide residues [16]. The final particle size falls within the range suitable for systemic circulation and BBB transcytosis [25], while the substantially reduced surface charge relative to unmodified PAMAM is expected to mitigate cytotoxicity.

### 3.3. Drug Loading and In Vitro Release

HPLC analysis yielded a drug loading capacity of 20.12% and an encapsulation efficiency of 71.78% for L57&PGP-PEG-PAMAM/Bre, achieved through physical encapsulation within PAMAM interior cavities, which is superior to the loading levels typically reported for dendrimer-based carriers in the glioma theranostics context [10].

The in vitro release profiles (Figure 10) exhibited a biphasic pattern comprising an initial burst phase followed by a sustained release phase. Free Bre displayed rapid dissolution, with nearly complete release within 10 h. In contrast, the PAMAM-based formulations demonstrated markedly prolonged release: PAMAM/Bre released 86.2% over 72 h, PAMAM-PEG/Bre released 58.9%, and L57&PGP-PEG-PAMAM/Bre released 54.6% over the same period. Each successive surface modification layer imposes an incremental steric diffusion barrier consistent with the PEG density-dependent release retardation mechanism previously documented for peptide-functionalized PEGylated carriers [11]. The progressively retarded release from PEGylated and dual-targeted formulations is attributed to the additional diffusion barrier imposed by the PEG corona and conjugated peptide layer, which restrict drug efflux from the dendrimer interior. This sustained release behavior is advantageous for maintaining therapeutic drug concentrations at the ischemic site over extended periods.

### 3.4. In Vivo Brain-Targeting Evaluation and Analysis

To directly compare the brain-targeting efficiency of the three formulations, whole-body fluorescence imaging was first performed at 1, 3 and 5 h post-intravenous injection (Figure 11A–C). Unmodified PAMAM/DiR showed negligible brain signal at all time points. PAMAM-PEG/DiR exhibited marginally enhanced but still faint cerebral fluorescence, attributable solely to prolonged circulation conferred by PEGylation. In contrast, L57&PGP-PEG-PAMAM/DiR displayed progressively intensifying brain fluorescence from 1 to 5 h, clearly distinguishable from both controls. Ex vivo organ imaging at 5 h (Figure 11D) confirmed predominant accumulation of the dual-targeted construct in the brain, with minimal off-target signal in peripheral organs.

Building on this comparative overview, the contribution of each targeting module was further dissected (Figure 12). Panel A shows the whole-body distribution of L57&PGP-PEG-PAMAM/DiR at 5 h, demonstrating strong cranial fluorescence. Panel B presents ex vivo brain fluorescence intensity quantification across all three groups, revealing that L57&PGP-PEG-PAMAM achieved approximately 4.2-fold higher brain signal than PAMAM-PEG and >15-fold higher than unmodified PAMAM. Panel C displays representative ex vivo images of excised brains from each group, visually corroborating the quantitative data. This marked enhancement arises from the sequential action of L57-mediated LRP1 transcytosis, which enables BBB traversal, followed by PGP-mediated CXCR2 anchoring, which prevents post-transcytosis washout-a synergistic mechanism absent in either single-component control.

Comparative imaging across the three formulations (Figure 12) clearly delineated the contribution of each targeting component. Unmodified PAMAM/DiR exhibited negligible brain fluorescence, indicating absence of intrinsic brain-targeting capability. PAMAM-PEG/DiR showed marginally enhanced brain signal, likely reflecting prolonged circulation time afforded by PEGylation. L57&PGP-PEG-PAMAM/DiR displayed substantially stronger brain fluorescence than both controls, confirming that the synergistic action of L57-mediated BBB transcytosis and PGP-mediated inflammatory site anchoring is essential for efficient brain-targeted delivery (see Supplementary Materials).

### 3.5. In Vivo Pharmacodynamic Efficacy

#### 3.5.1. Neurological Function

Neurological scoring 24 h post-reperfusion (Figure 13) revealed that MCAO rats exhibited severe functional deficits (score: 4.17 ± 0.39; *p* < 0.01 vs. Sham). Free Bre treatment significantly improved neurological function (2.76 ± 0.41; *p* < 0.01 vs. MCAO). The L57&PGP-PEG-PAMAM/Bre group achieved the lowest deficit score (1.52 ± 0.28; *p* < 0.01 vs. Bre), indicating that targeted nanocarrier delivery substantially augmented the neuroprotective effect of Bre.

#### 3.5.2. Cerebral Infarct Volume

TTC staining (Figure 14) revealed extensive white (infarcted) regions in MCAO rat brains, with an infarct volume of 47.6 ± 8.9% (Figure 15; *p* < 0.01 vs. Sham). Free Bre reduced infarct volume to 34.8 ± 7.2% (*p* < 0.01 vs. MCAO). The L57&PGP-PEG-PAMAM/Bre formulation produced the most pronounced reduction, decreasing infarct volume to 21.7 ± 6.4% (*p* < 0.01 vs. Bre), representing a 54.4% reduction relative to untreated MCAO rats.

#### 3.5.3. Histopathological Analysis

H&E staining (Figure 14) provided histological corroboration of the therapeutic outcomes. Sham-operated rats displayed normal neuronal architecture with orderly arrangement, clear structural definition, and no interstitial edema (Figure 16A). MCAO rats exhibited pronounced ischemia–reperfusion injury at 24 h, including meningeal edema and thickening, inflammatory cell infiltration, mild cortical and glial cell proliferation, and focal disruption of the neuropil within the cerebral cortex (Figure 16B). Free Bre treatment partially attenuated these pathological changes, with reduced meningeal edema, diminished inflammatory infiltration, and partial restoration of neuropil integrity (Figure 16C). The L57&PGP-PEG-PAMAM/Bre group showed further improvement across all histopathological parameters, with neuronal morphology and tissue architecture closely approaching those of the Sham group (Figure 16D).

#### 3.5.4. Anti-Inflammatory Effects

Quantification of pro-inflammatory cytokines in brain tissue homogenates (Figure 17) revealed that MCAO significantly elevated IL-15 (87.92 ± 11.49 pg/mL) and IL-27 (221.78 ± 43.25 pg/mL) relative to Sham levels (20.76 ± 5.63 and 72.64 ± 13.97 pg/mL, respectively; *p* < 0.01). Free Bre reduced IL-15 to 65.69 ± 7.38 pg/mL and IL-27 to 137.59 ± 36.57 pg/mL (*p* < 0.01 vs. MCAO). The L57&PGP-PEG-PAMAM/Bre formulation achieved the most pronounced suppression, lowering IL-15 to 33.74 ± 5.91 pg/mL and IL-27 to 93.46 ± 16.91 pg/mL (*p* < 0.01 vs. Bre). These results indicate that targeted delivery of Bre to the ischemic inflammatory microenvironment substantially amplifies its anti-inflammatory action, representing a key mechanism underlying the enhanced neuroprotection.

#### 3.5.5. Discussion of In Vivo Pharmacodynamic Outcomes in the Context of Nanoparticle-Based Stroke Therapy

The pharmacodynamic gains observed here warrant comparison with recent nanoparticle-based interventions in ischemic stroke models. Xia et al. [23] reported a BBB-penetrating, microglia-targeted, hypoxia-responsive nanogel that reduced infarct volume by approximately 40% relative to untreated MCAO rats after intravenous administration; in the present study, the L57&PGP-PEG-PAMAM/Bre formulation achieved a 54.4% infarct reduction, a comparable or somewhat larger effect despite oral gavage delivery. He et al. [7] reviewed nanomaterial-based drug delivery systems for ischemic stroke and noted that most BBB-crossing carriers rely on a single targeting mechanism, which limits post-barrier retention at the lesion. The sequential LRP1 transcytosis followed by CXCR2 neutrophil anchoring employed here addresses this limitation directly, as reflected in the sustained brain fluorescence signal and the superior suppression of IL-15 and IL-27 relative to free Bre. Wang et al. [10] demonstrated that BBB-crossing PAMAM dendrimers functionalized for glioma theranostics achieved brain accumulation sufficient for imaging and therapy; the present data extend this concept by showing that the same dendrimer platform, when equipped with a dual-ligand sequential targeting architecture, can deliver a small-molecule neuroprotectant to the ischemic penumbra with measurable functional and histological benefit. It should be noted that the oral administration route used in this study likely results in lower absolute brain bioavailability than intravenous injection employed in most BBB-crossing nanoparticle studies; the fact that significant neuroprotection was nonetheless achieved underscores the efficiency of the dual-targeting mechanism. Future pharmacokinetic studies quantifying brain drug concentrations after intravenous versus oral dosing would further clarify the contribution of each targeting step.

## 4. Conclusions

A dual-targeting nanocarrier, L57&PGP-PEG-PAMAM, was successfully constructed by sequentially conjugating the BBB-penetrating peptide L57 and the inflammatory site-targeting peptide PGP onto PEGylated fifth-generation PAMAM dendrimers. The nanocarrier exhibited a hydrodynamic diameter of 140.2 ± 0.6 nm, a zeta potential of 9.32 mV, and efficient Bre encapsulation (DL: 20.12%; EE: 71.78%) with sustained release kinetics (54.6% over 72 h). In vivo fluorescence imaging confirmed substantially enhanced brain accumulation relative to non-targeted and PEGylated controls, attributable to the sequential LRP1-mediated BBB transcytosis and CXCR2-mediated neutrophil anchoring. In MCAO rats, the Bre-loaded nanocarrier reduced infarct volume to 21.7 ± 6.4%, improved neurological scores to 1.52 ± 0.28, and suppressed IL-15 and IL-27 levels to 33.74 and 93.46 pg/mL, respectively—all significantly superior to free Bre treatment. The enhanced efficacy is mechanistically linked to the dual-targeting architecture that concentrates Bre at the ischemic inflammatory lesion. Future investigations will address the in vivo degradation and clearance pathways of the PAMAM-based carrier, elucidate the molecular mechanisms governing its anti-inflammatory effects, and evaluate combinatorial strategies with thrombolytic therapy to further broaden its translational potential.

## Figures and Tables

**Figure 1:**
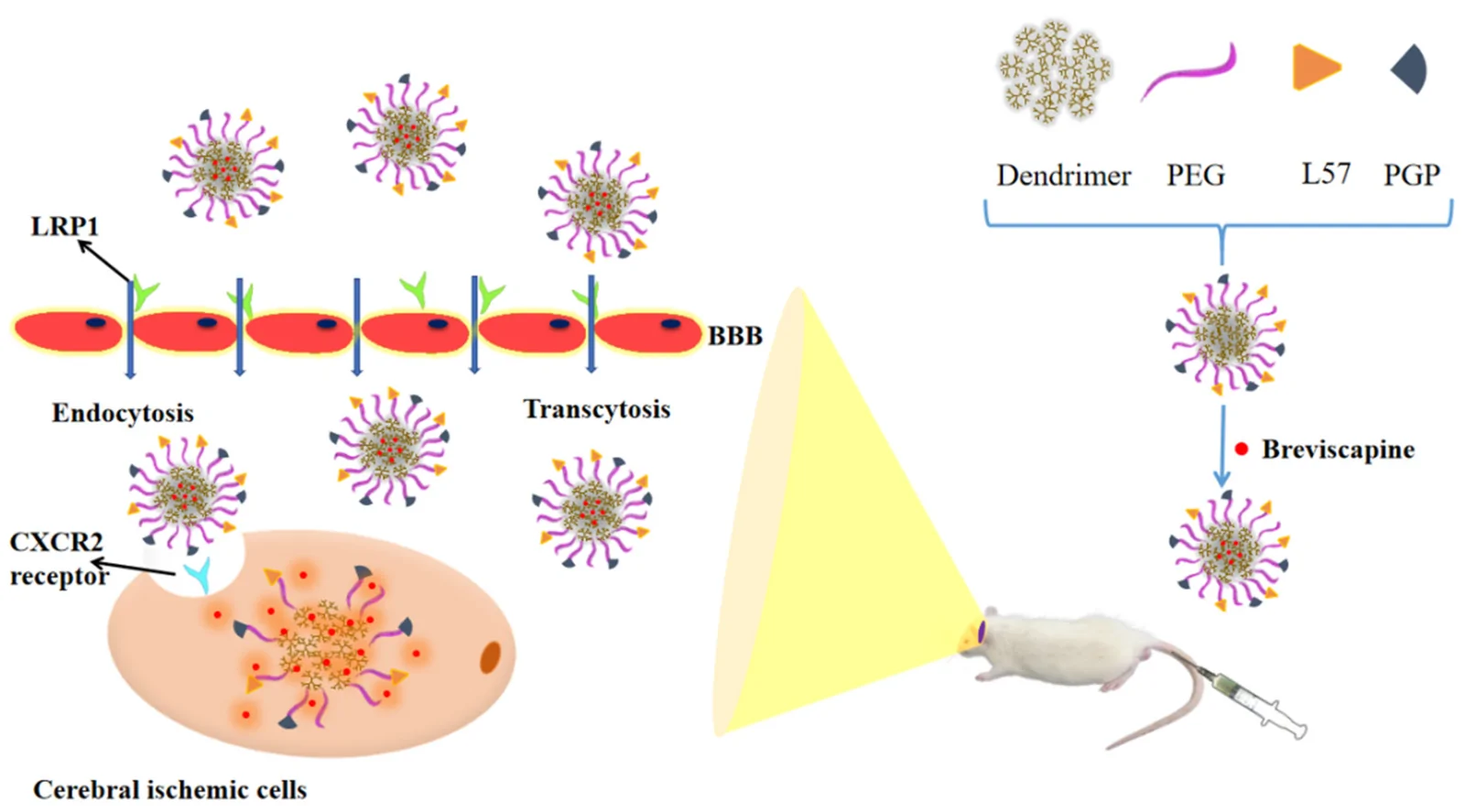
Schematic diagram of dual-targeting dendrimer nano drug system to delivery breviscapine to the inflammatory sites of the ischemic brain for treatment of ischemic stoke.

**Figure 2:**
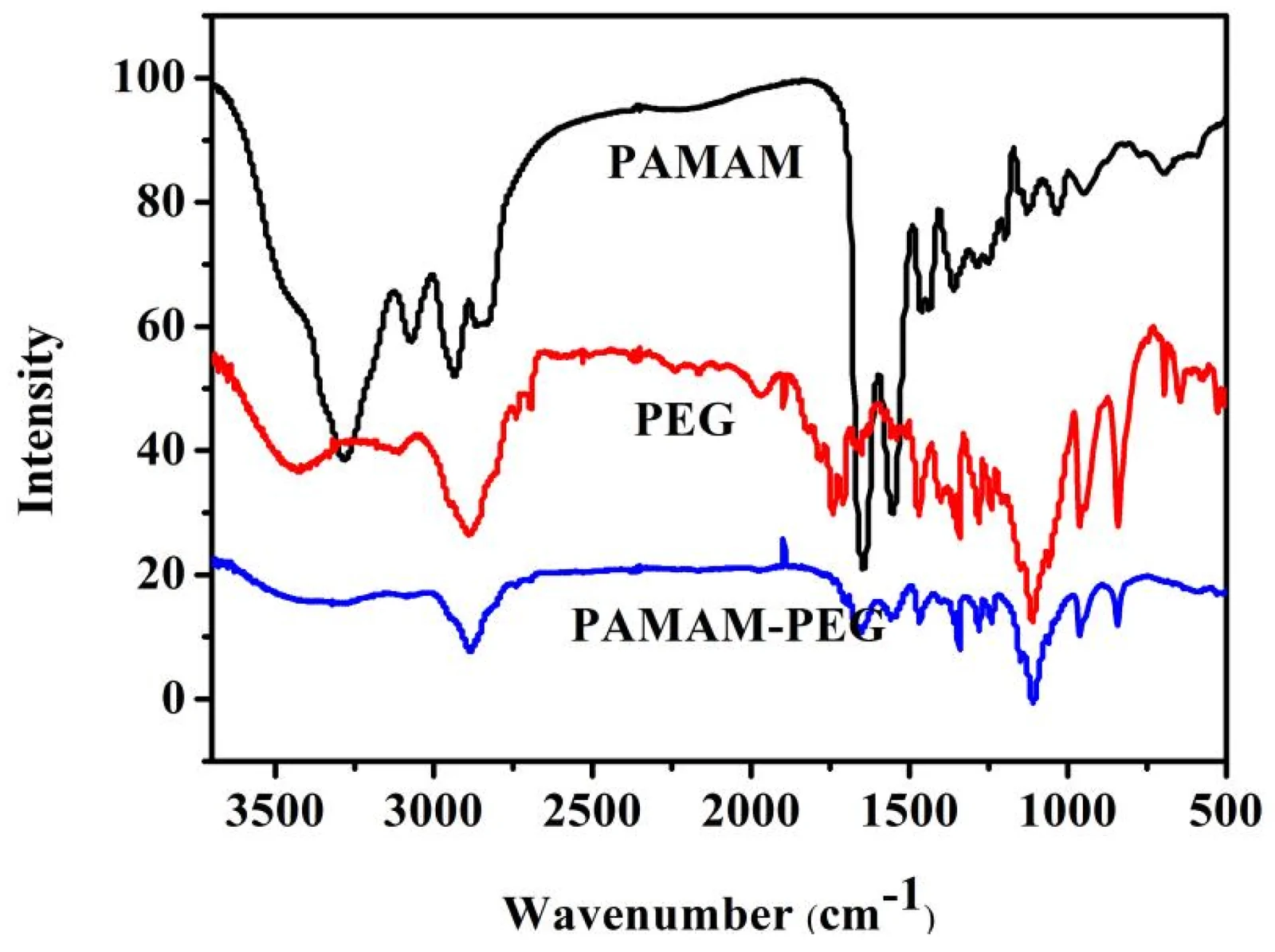
FT-IR spectrum of PAMAM, PEG and PAMAM-PEG.

**Figure 3:**
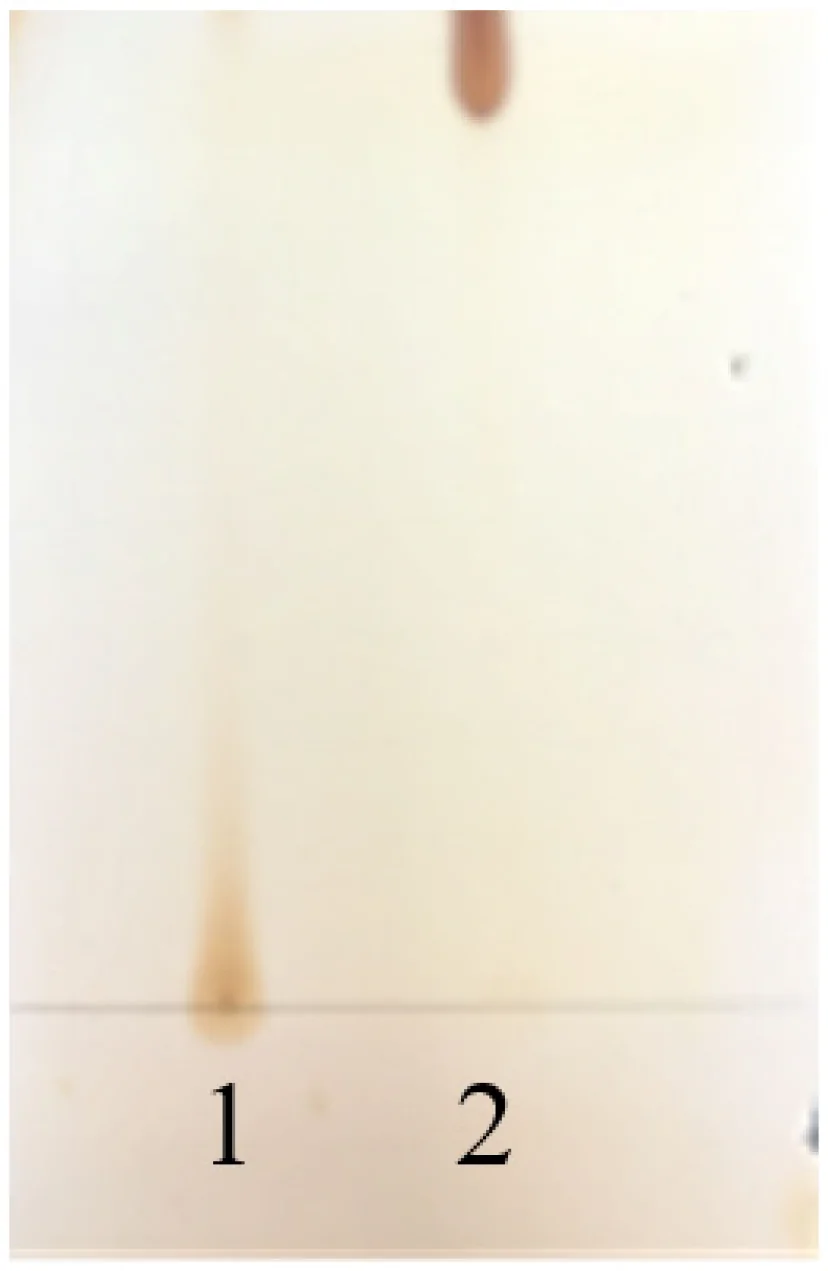
PAMAM-PEG thin-layer chromatogram 1. PAMAM-PEG 2. PEG.

**Figure 4:**
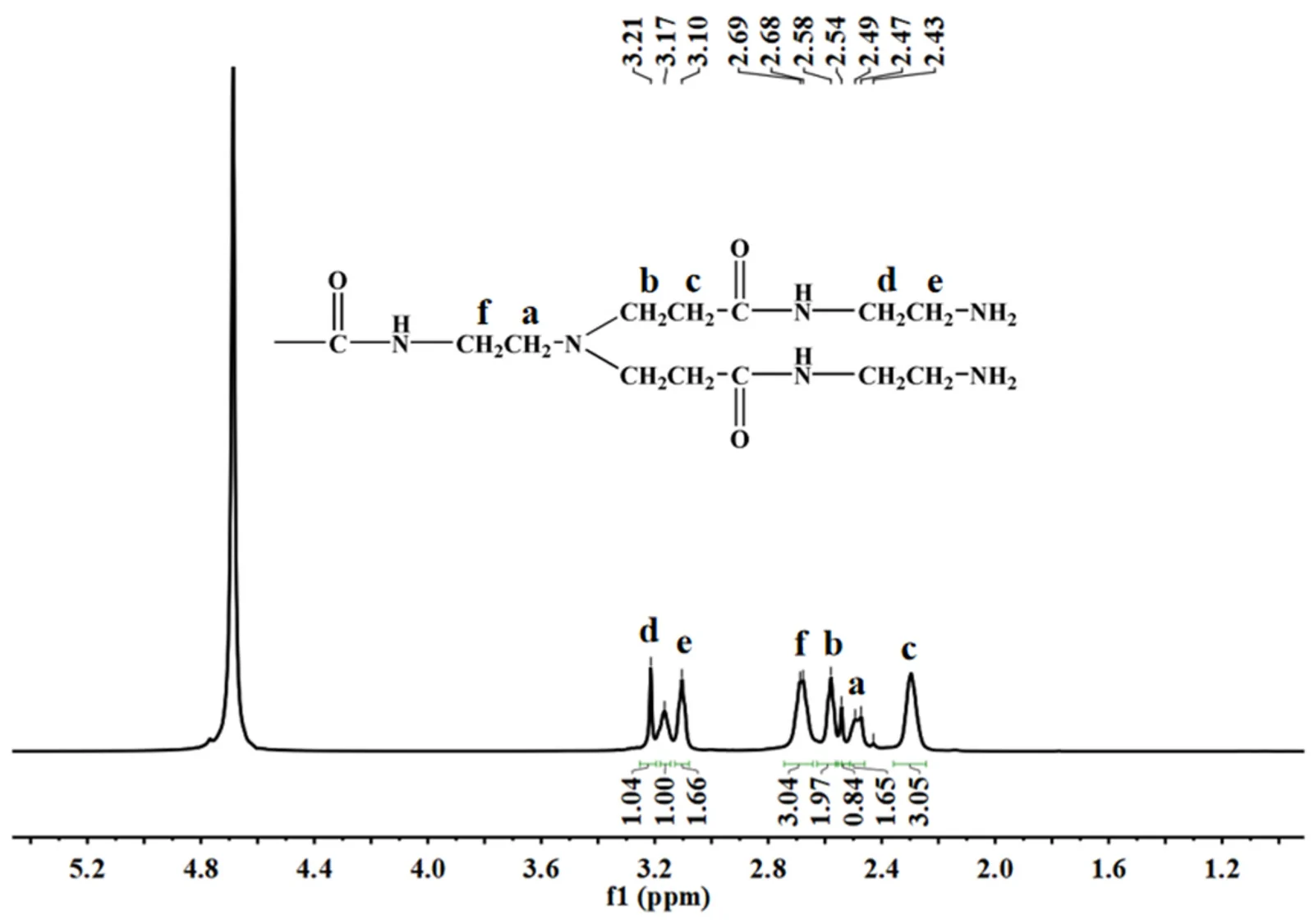
G 5.0 PAMAM ^1^H-NMR spectrum.

**Figure 5:**
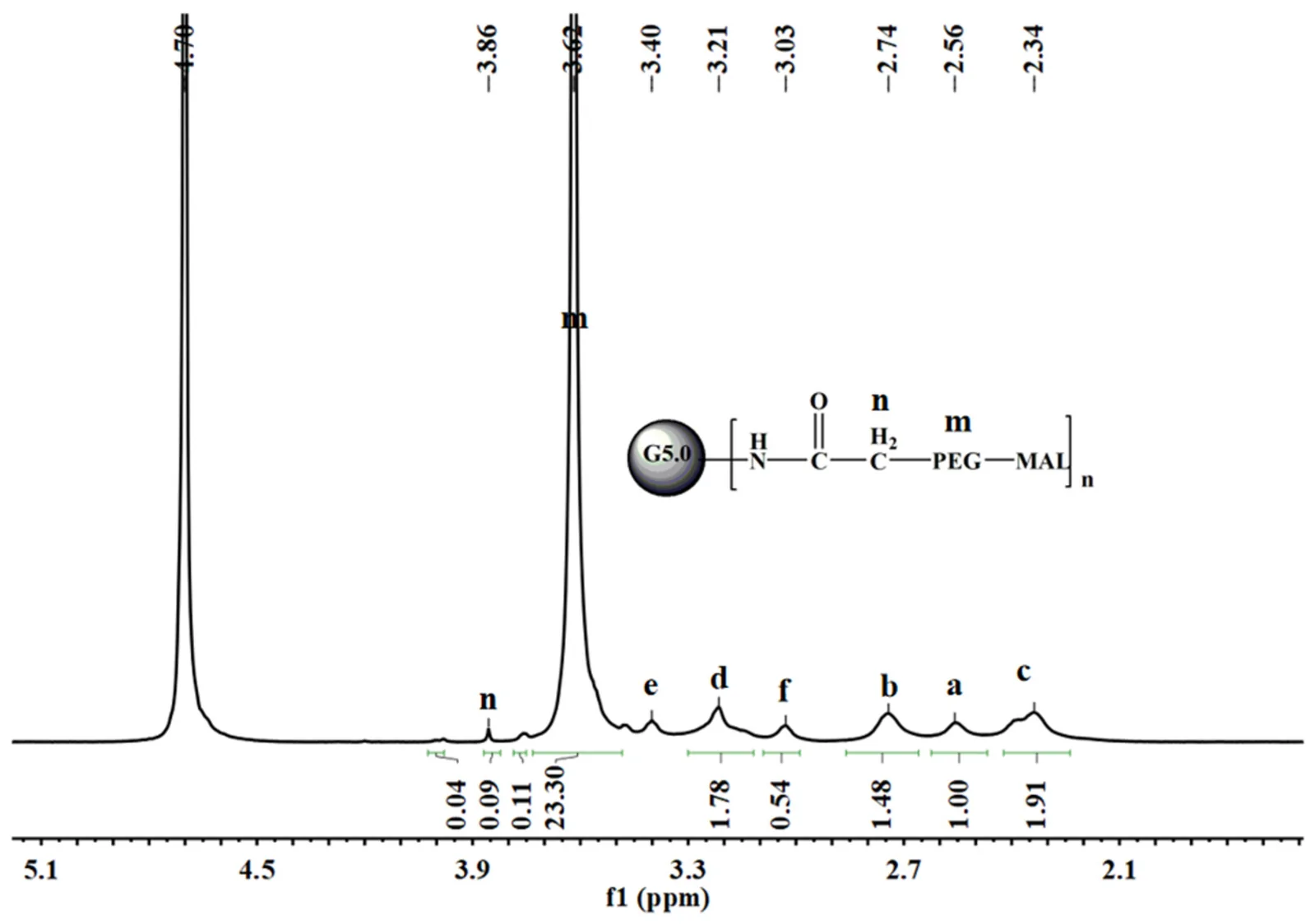
G 5.0 PAMAM-PEG ^1^H-NMR spectrum.

**Figure 6:**
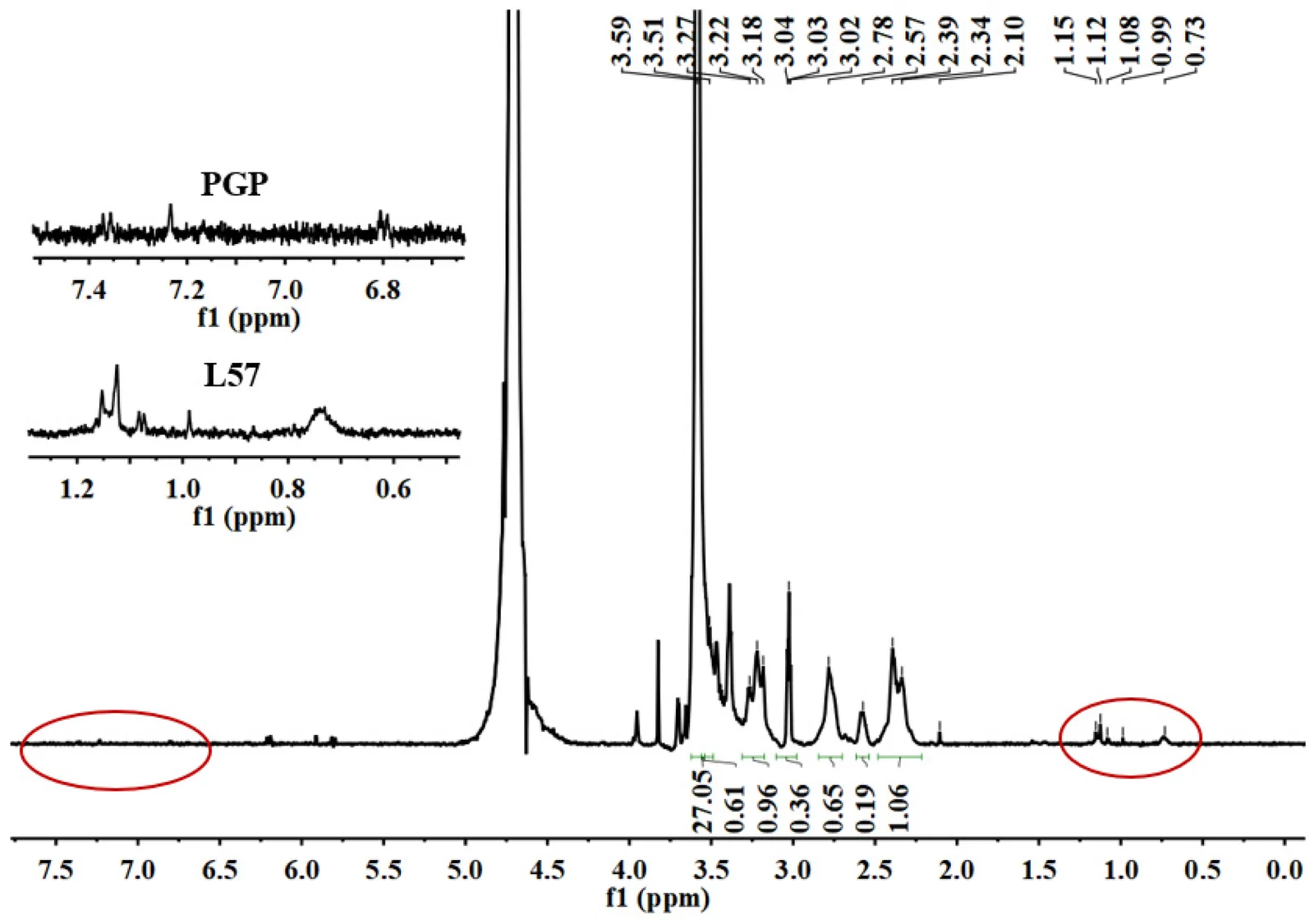
L57&PGP-PEG-PAMAM ^1^H-NMR spectrum.

**Figure 7:**
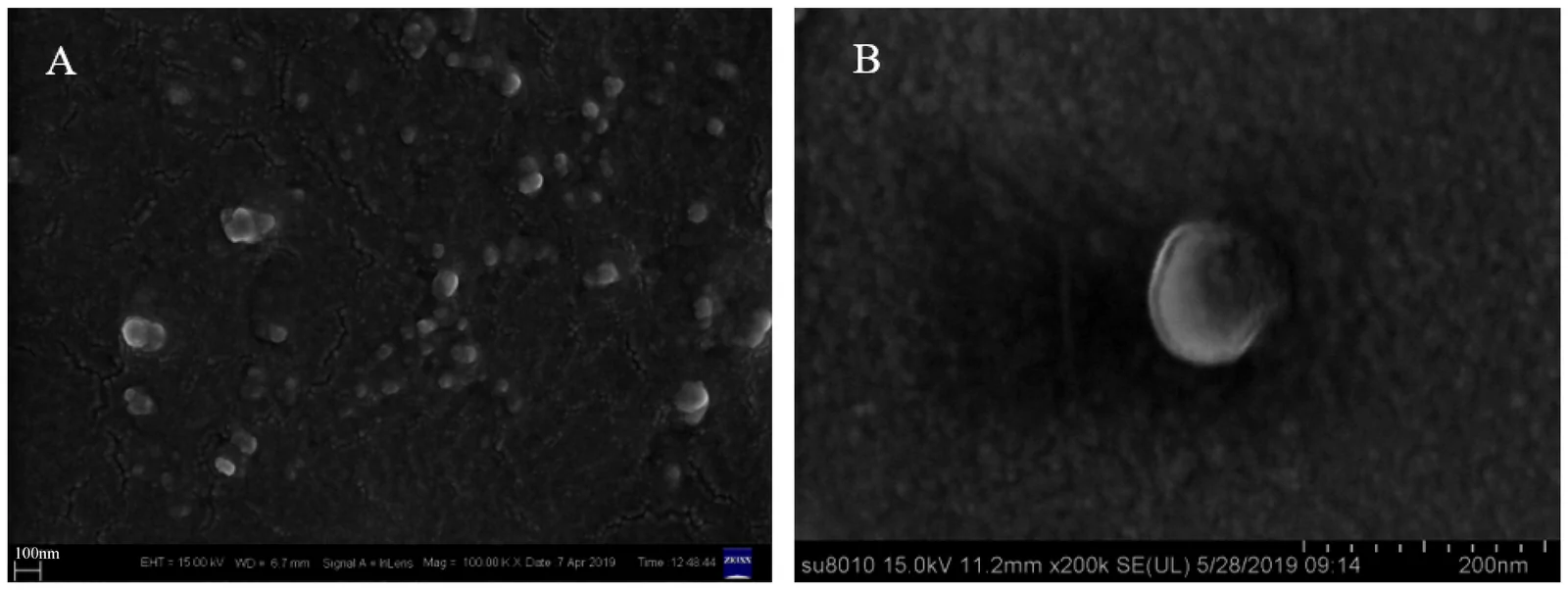
Scanning electron microscope image of L57&PGP-PEG-PAMAM NPs.

**Figure 8:**
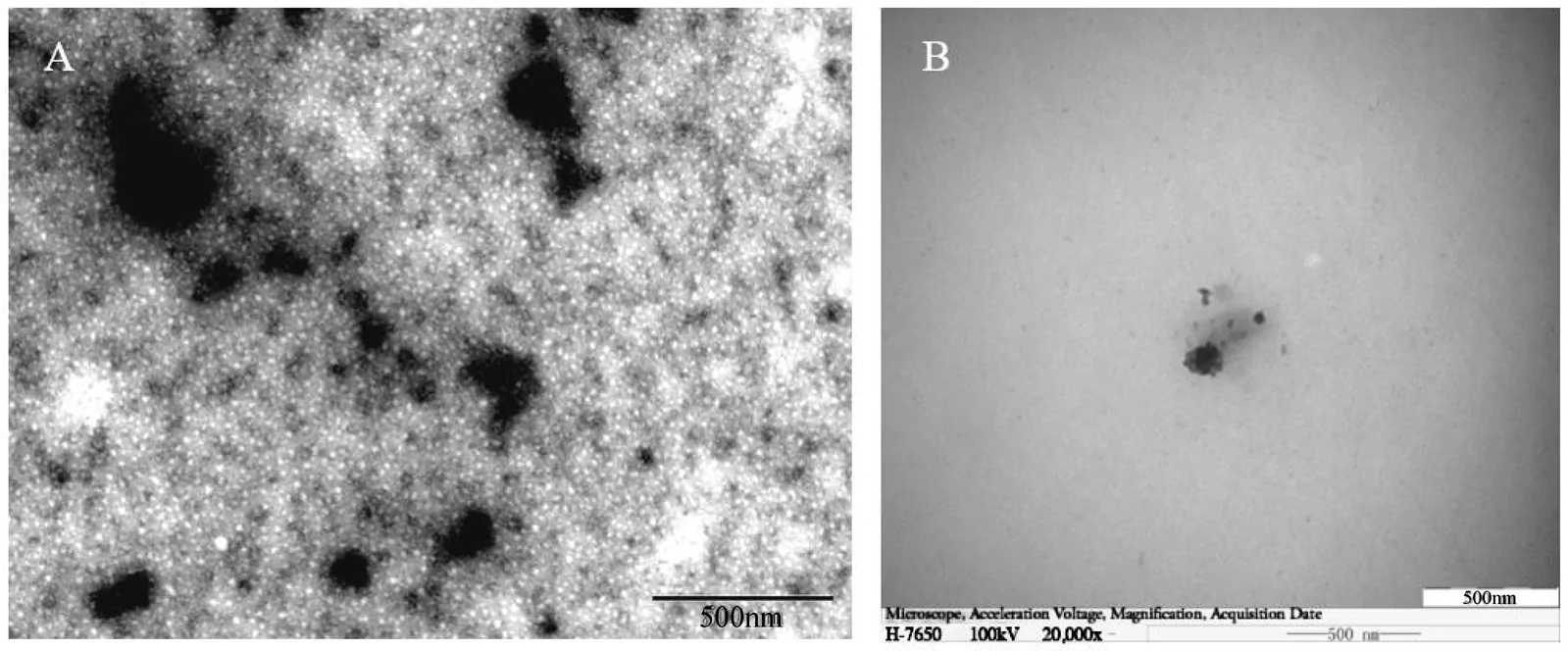
Transmission electron microscope image of L57&PGP-PEG-PAMAM NPs.

**Figure 9:**
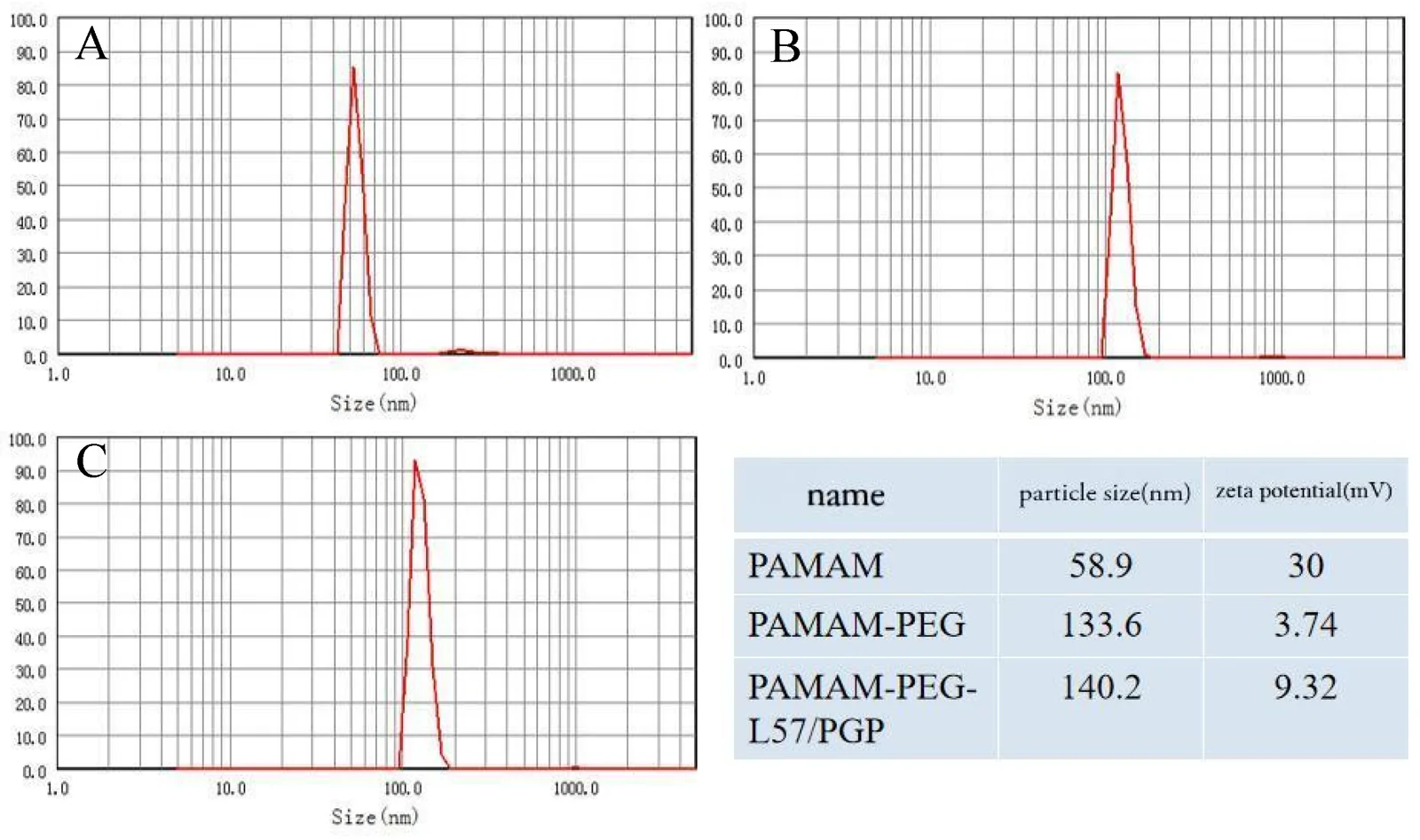
Particle size distribution and potential values of PAMAM (**A**), PAMAM-PEG (**B**), L57&PGP-PEG-PAMAM (**C**).

**Figure 10:**
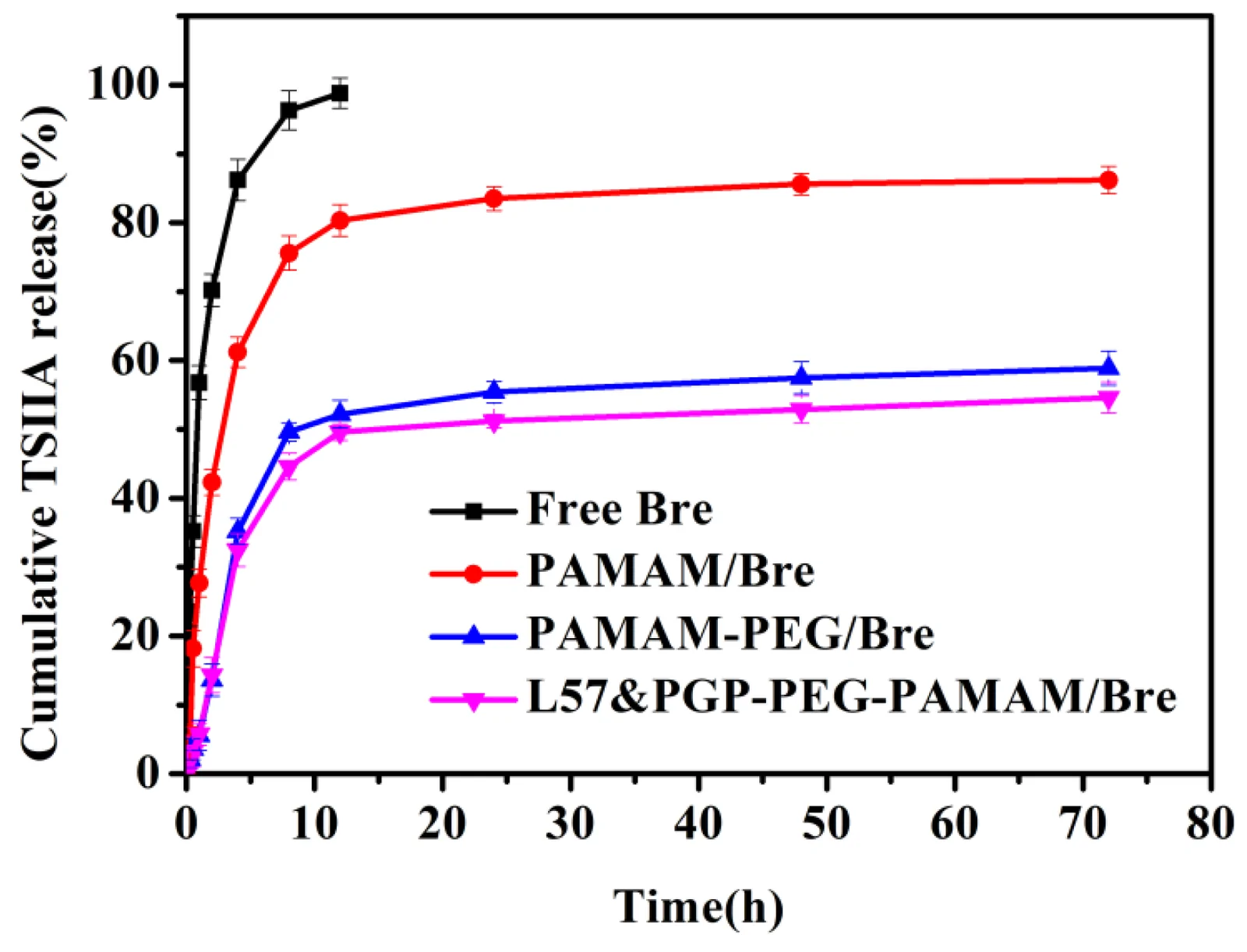
In vitro release profiles of breviscapine and three PAMAM drug-loading systems.

**Figure 11:**
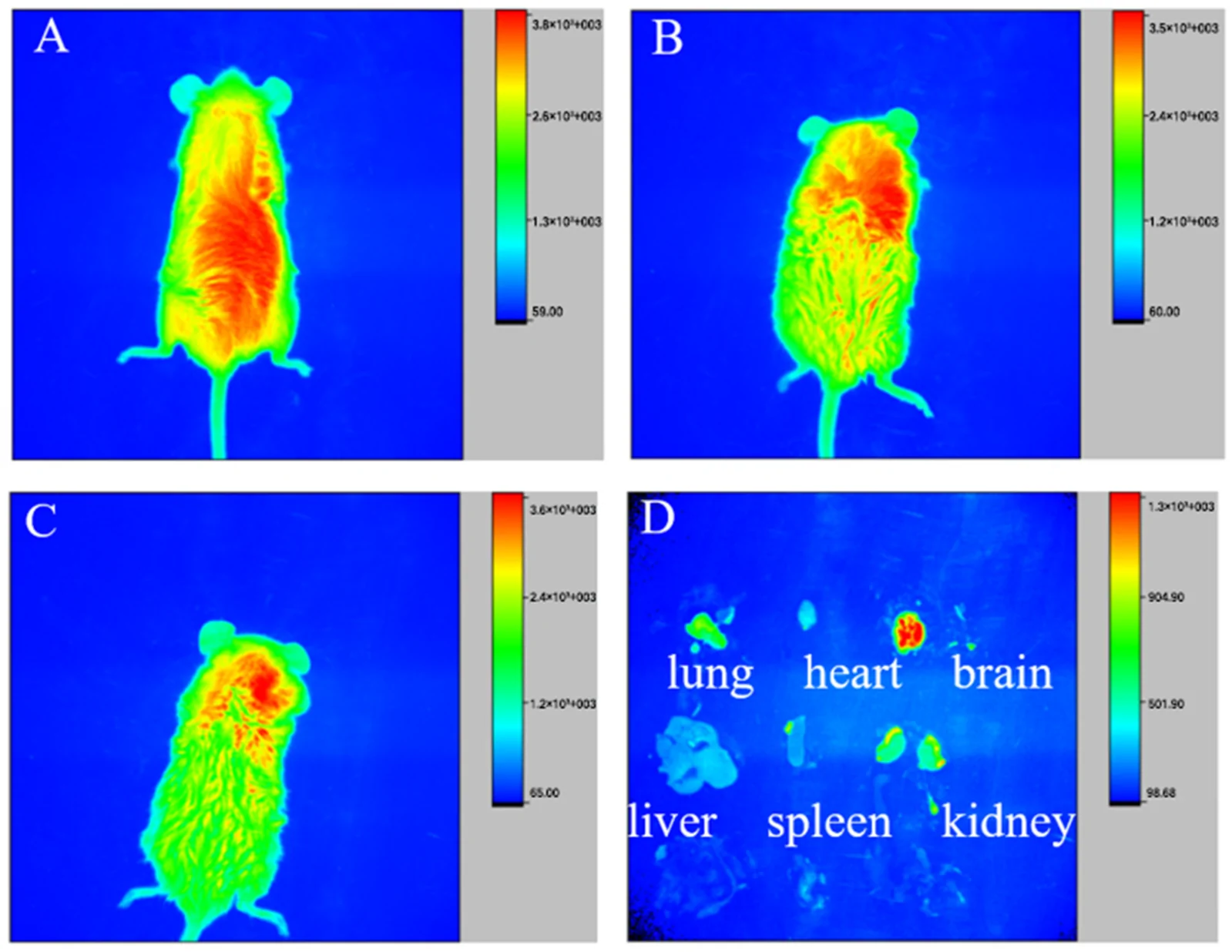
Real-time dynamic distribution of brain targeting of L57&PGP-PEG-PAMAM nanocarriers within five hours ((**A**) observed 1 h after injection; (**B**) observed 3 h after injection; (**C**) observed 5 h after injection; (**D**) fluorescence distribution of main organs after dissection of mice 5 h after injection).

**Figure 12:**
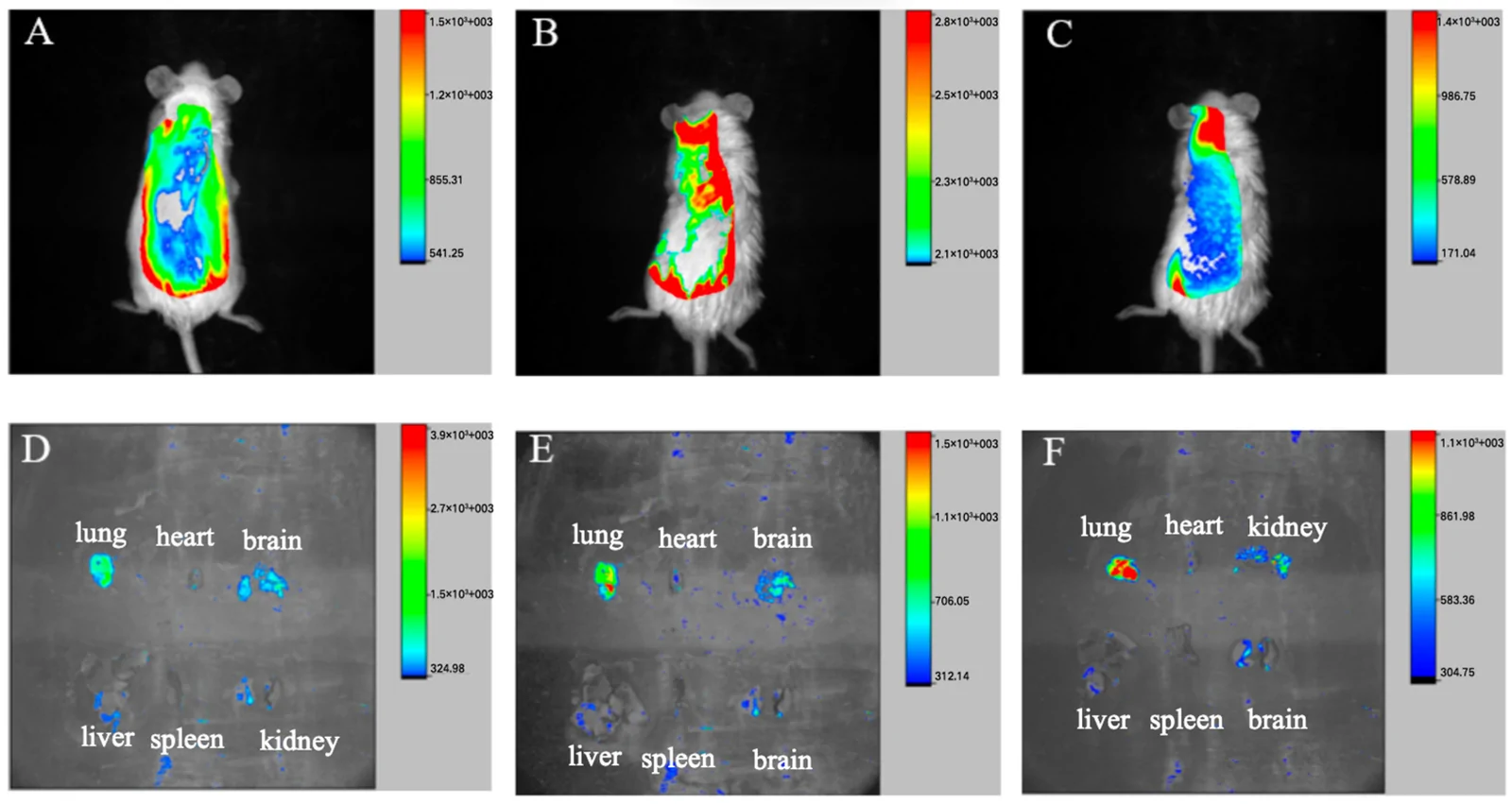
Comparison of brain targeting performance of PAMAM, PAMAM-PEG, and L57&PGP-PEG-PAMAM, three composite materials. From left to right, the images show the in vivo distribution of three composites, PAMAM (**A**,**D**), PAMAM-PEG (**B**,**E**), and L57&PGP-PEG-PAMAM (**C**,**F**), in mice at a certain time after intravenous injection.

**Figure 13:**
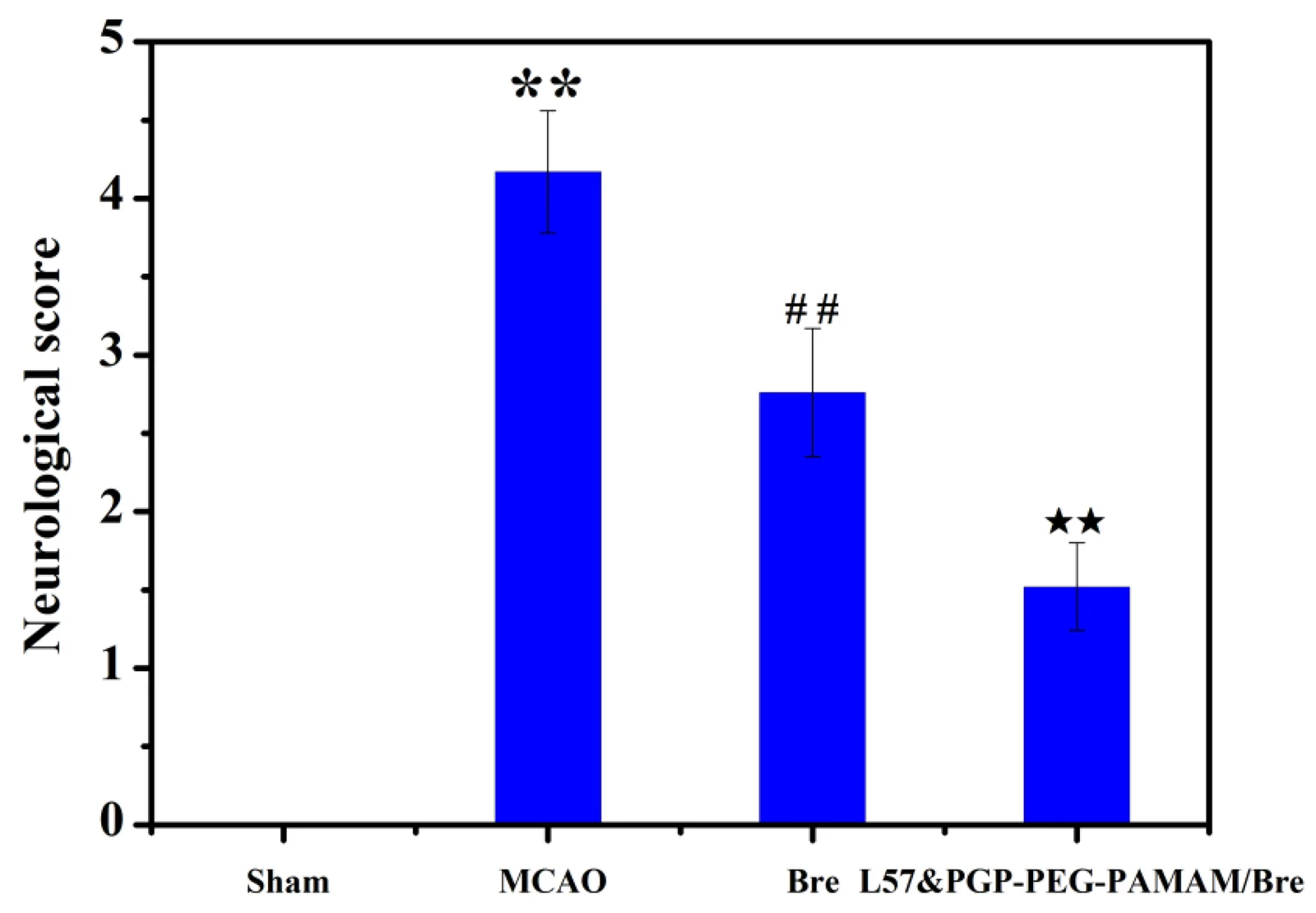
Effects of four groups of SD rats on neurological function 24 h after surgery.

**Figure 14:**
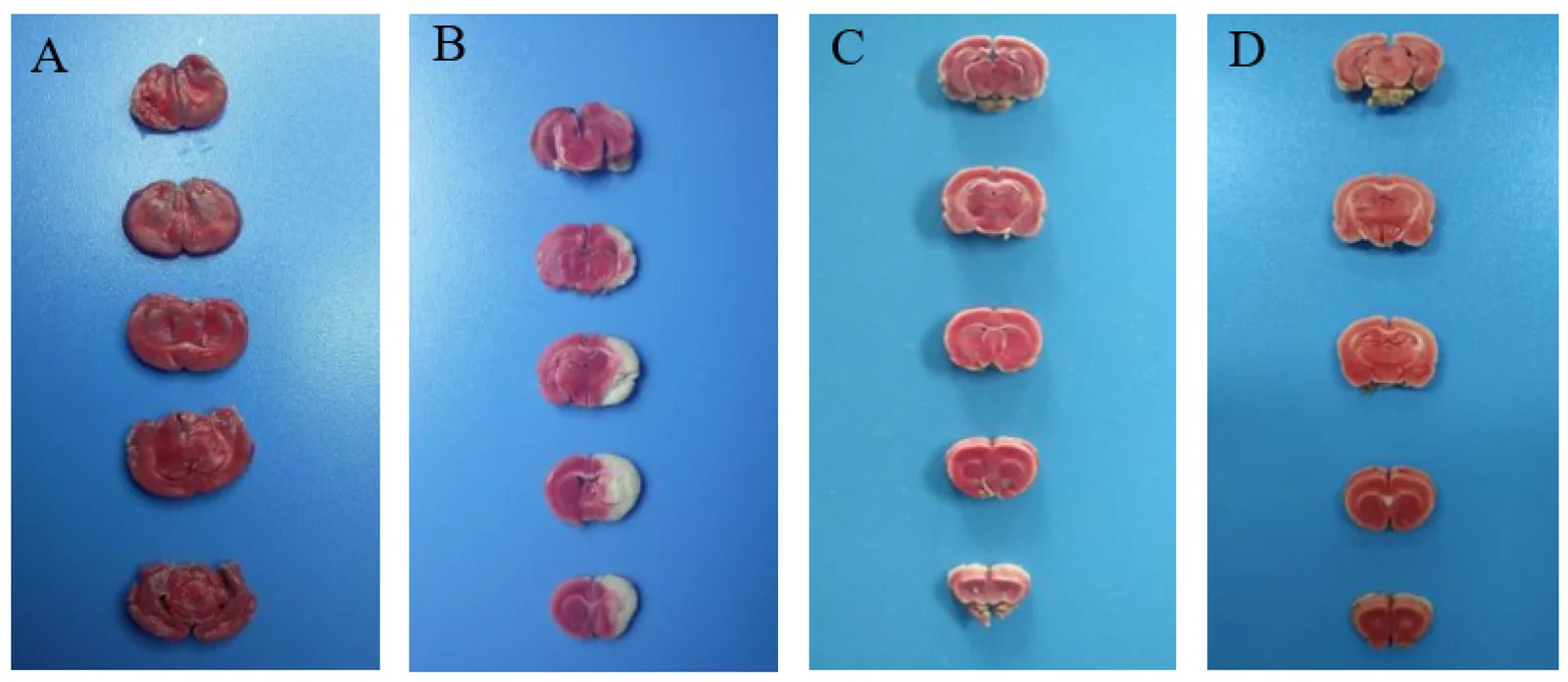
TTC staining for brain slices of SD rats after dosing of four groups, where (**A**) represents the Sham group, (**B**) the MCAO group, (**C**) the bulk drug group, and (**D**) the L57&PGP-PEG-PAMAM/Bre group.

**Figure 15:**
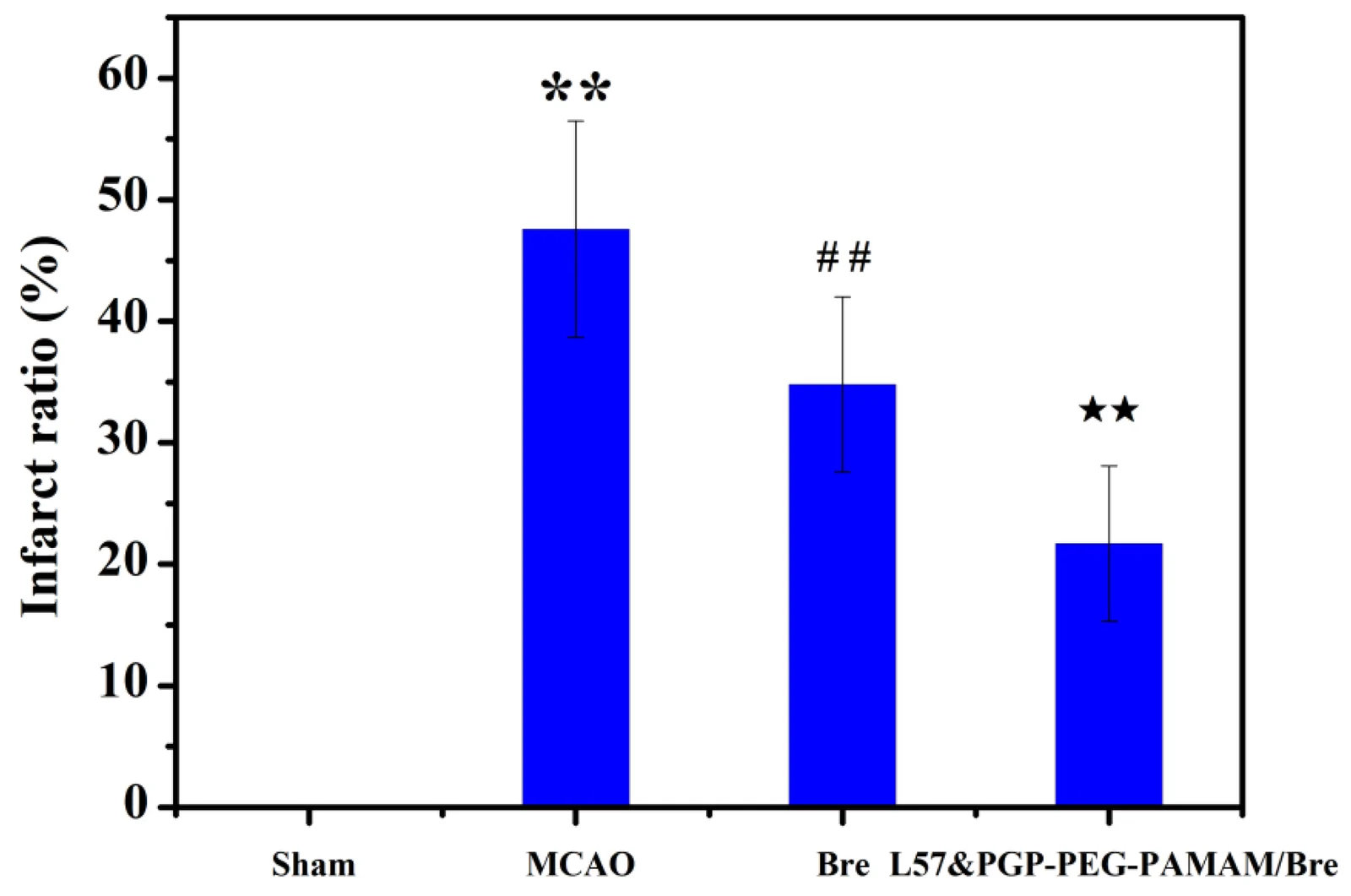
Area of cerebral infarction after administration in four groups of SD rats.

**Figure 16:**
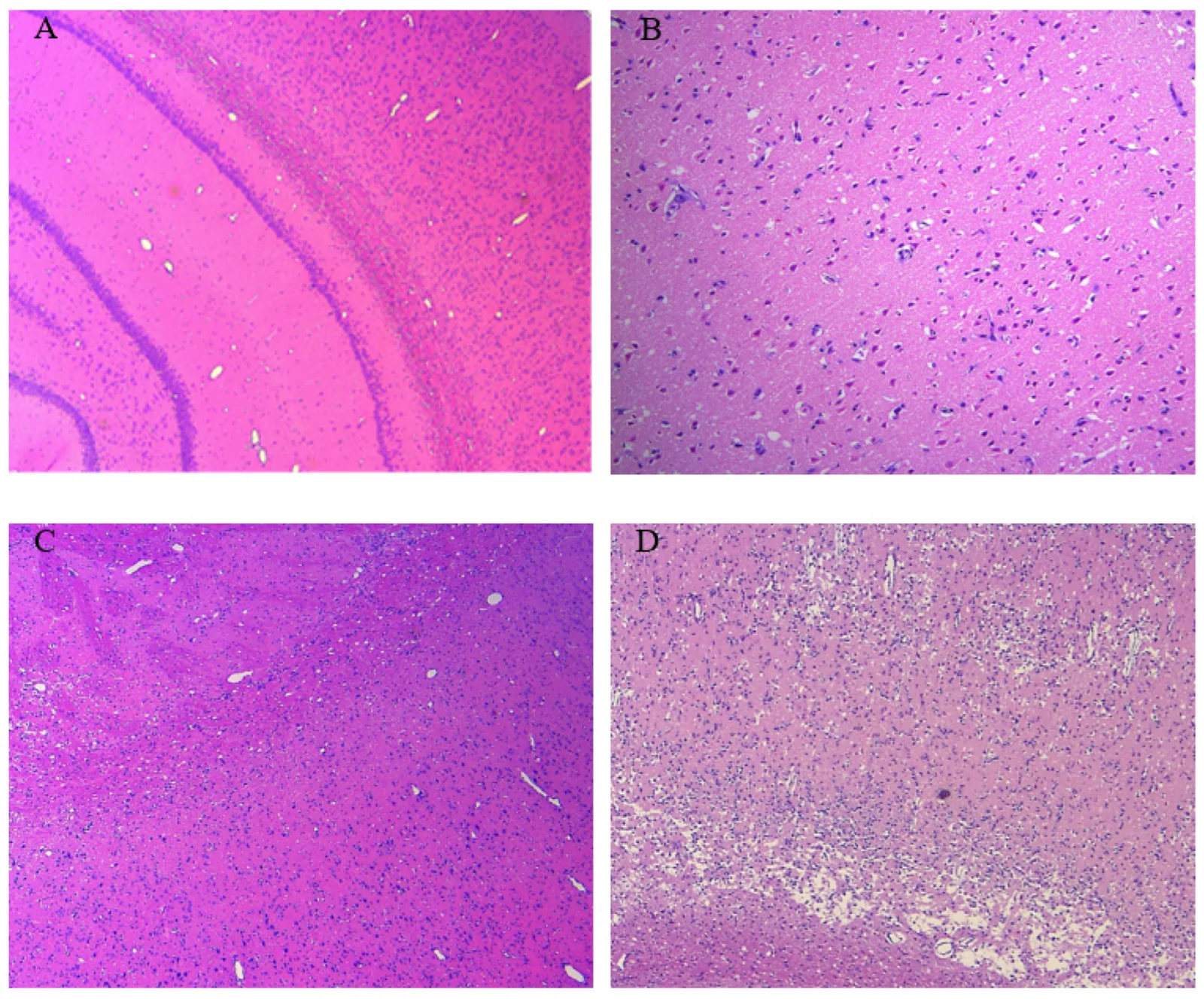
Effect of four groups of experimental rats on brain pathology after administration. Panel (**A**) represents the Sham group, Panel (**B**) the model group, Panel (**C**) the breviscapine group, and Panel (**D**) the L57&PGP-PEG-PAMAM/Bre group.

**Figure 17:**
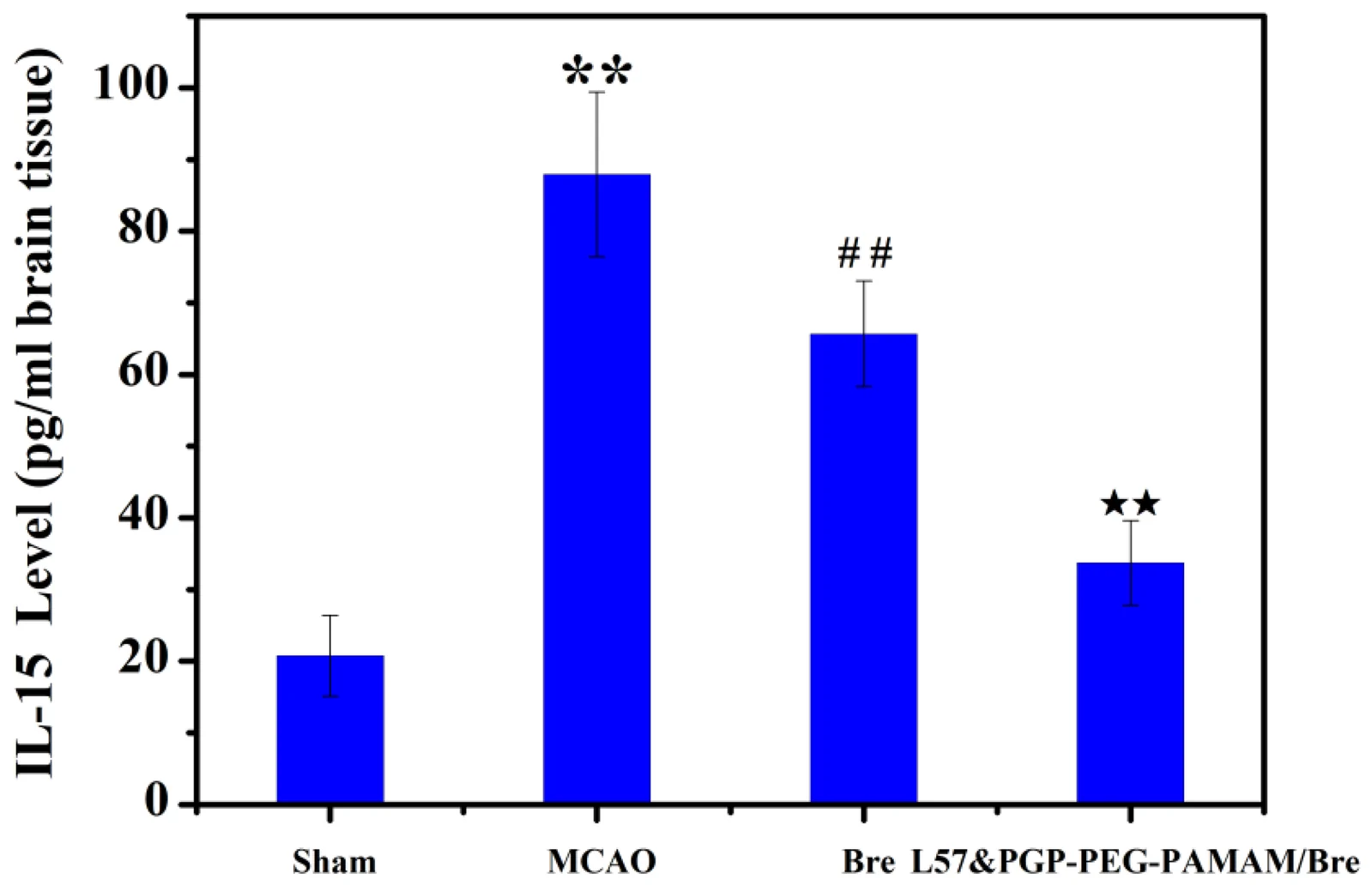
IL-15 level in four groups of SD rats after administration.

## Data Availability

The datasets used and/or analyzed during the current study are available from the corresponding author on reasonable request.

## Supplementary Materials

The following supporting information can be downloaded at: https://www.mdpi.com/article/10.3390/molecules31193363/s1, Table S1:The main instrument, model and manufacturer of the experiment; Table S2: Experimental reagents, purity and factory; Table S3: The main instrument, model and manufacturer of the experiment; Table S4; Experimental reagents, purity and factory; Table S5: Peak area, concentration and RSD value of breviscapine measured six times; Table S6: Method recovery of breviscapine; Table S7: The main instrument, model and manufacturer of the experiment; Table S8: Experimental reagents, purity and factory; Figure S1: UV absorption spectrum of breviscapine solution; Figure S2: Breviscapine standard curve; Figure S3: Chromatogram of breviscapine; Figure S4: Chromatogram of L57&PGP-PEG-PAMAM; Figure S5: Nerve function scoring criteria.
