# Supplementary material for: Cross-and-Capture: A Dual-Ligand PAMAM Nanocarrier for Sequential BBB Penetration and Ischemic Lesion Retention of Breviscapine
Source: Molecules. 2026 Sep 22;31(19):3363. doi: 10.3390/molecules31193363 (PMC13666067; doi:10.3390/molecules31193363)
Supplement: Supplementary file 1 [file molecules-31-03363-s001.zip › molecules-4530902-supplementary.pdf]

Supporting information

Cross-and-Capture: A Dual-Ligand PAMAM Nanocarrier for Sequential BBB Penetration and Ischemic Lesion Retention of Breviscapine

Haoran Guo [†], Dandan Han [†] and Xin Liu *

School of Materials Science and Chemical Engineering, Harbin University of Science and Technology, Harbin 150040, China; 19984187566@163.com (H.G.); 18845613098@163.com (D.H.)

* Correspondence: xinliu98@126.com

† These authors contributed equally to this work.

1. Preparation of nanocarrier materials and Apparatus

1.1 Experimental Instruments and Equipment

Table S1 The main instrument, model and manufacturer of the experiment

InstrumentName	Model	Manufacturer
Vacuum drying oven	ZK072	Shanghai Experimental Instruments Factory Co., Ltd
Vacuum freeze-dryer	SCIENTZ-12N	Xinzhi Technology Co., Ltd
Magnetic Stirrer	CL-200	Gongyi Yuhua Instrument Co., Ltd
Vacuum oil pump	2XZ-4	Shanghai Lichen Technology Co., Ltd
Tabletop high-speed centrifuge	TG1XZ6-WS	Xiangyi Instrument Development Co., Ltd
Rotary evaporator	RE-52A	Shanghai Yarong Biochemical Instrument Factory
pH meter	pH610	WIGGENS Germany
Electronic analytical balance	SECURA213-1CN	Sartorius Group

InstrumentName	Model	Manufacturer
infrared spectrometer	T60SXBFTIR 型	Nicole Company of the United States
Scanning electron microscope	JSM-5610LV	Japan Electronic Co., Ltd
Transmission electron microscope	SUPRA 55	Carl Zeiss AG
Nuclear Magnetic Resonance Spectrometer	AVANCEIIIHD500MHz	Brook Scientific Instruments Co., Ltd
Nanoparticle Size and Zeta Potential Analyzer	BT-90 Nano Laser Particle Size Analyzer	Dandong Bait Instrument Co., Ltd

1.2 Experimental Reagents

Table S2 Experimental reagents, purity and factory

Reagent name	Specification	manufacturer
NHS-PEG-MAL	Mr=2000	Shanghai Fanshuo Biotechnology Co., Ltd
anhydrous methanol	Analytical Reagent	Bailingwei Technology Co., Ltd
ethylenediamine	Analytical Reagent	Shanghai McLean Biochemical Technology Co., Ltd
methyl acrylate	Analytical Reagent	Shanghai Aladdin Biochemical Technology Co., Ltd
Thin-layer silica gel	GF254	Qingdao Jiyida Silicone Reagent Co., Ltd
anhydrous ether	Analytical Reagent	Shanghai Aladdin Biochemical Technology Co., Ltd

2. Performance study

2.1 Materials and Apparatus

2.1.1 Experimental Instruments and Equipment

Table S3 The main instrument, model and manufacturer of the experiment

InstrumentName	Model	Manufacturer
Magnetic Stirrer	DF-101S	Xi'an Changyi Instrument Equipment Co., Ltd
Electronic analytical balance	SECURA213-1CN	Sartoris Group, Germany
Nuclear magnetic resonance spectrometer	AVANCEIIIHD500MHz	Bruker Corporation
Constant temperature blast drying oven	DHG-9070A	Shanghai Yiheng Scientific Instrument Co., Ltd
Vacuum oil pump	2XZ-4	Shanghai Lichen Technology Co., Ltd
Vacuum freeze dryer	SCIENTZ-12N	Xinzhi Biotechnology Co., Ltd
PH meter	pH610	WIGGENS Germany
Desktop high-speed centrifuge	TG16-WS	Xiangyi Laboratory Instrument Development Co., Ltd
Fluorescence live imaging device	IS4000MM	Bruker BioSpin Corporation, USA
High performance liquid chromatography analyzer	UltiMate3000	Thermo Fisher Scientific Co., Ltd
ultrafiltration tube	MD=30000	
UV visible spectrophotometer	T6 New Century	Thermo Fisher Scientific Co., Ltd

2.1.2 Experimental Reagents

Table S4 Experimental reagents, purity and factory

Reagent name	Specification	Manufacturer
chloral hydrate	Analytical Reagent	Qingdao Yulong Seaweed Co., Ltd
Dimethyl sulfoxide	Analytical Reagent	Bailingwei Technology Co., Ltd

Reagent name	Specification	Manufacturer
(DMSO)		
ICR mice	22 g	Shanghai Slack Laboratory Animal Co., Ltd
Lamplighter extract	Analytical Reagent	Nanjing Chunqiu Bioengineering Co., Ltd
DiR fluorescent dye	98%	Tianjin Baiying Biotechnology Co., Ltd
Chromatographic methanol	Chromatographic purity	Sigma
medical alcohol	95 degrees	Yongchang

2.2 Experimental Section

2.2.1 Methodological Evaluation

(1) Determination of Detection Wavelength

Weigh 10 mg of breviscapine into a 50 mL volumetric flask and dilute it to volume with methanol to prepare a breviscapine stock solution with a concentration of 0.2 mg·mL^{-1}. Subsequently, transfer 0.5 mL, 1 mL, and 2 mL of this stock solution into three separate 10 mL volumetric flasks, respectively, and dilute each to volume to obtain breviscapine standard solutions with concentrations of 10.0 mg·mL^{-1}, 20 mg·mL^{-1}, and 40 mg·mL^{-1}, respectively. Utilize a UV spectrophotometer to measure the characteristic absorption peaks of these three samples, with each sample being measured three times to calculate the mean value, thereby determining the position of the characteristic absorption peak for breviscapine.

(2) Standard Curve Accurately

weigh 10 mg of scutellarin reference standard into a 50 mL volumetric flask and dilute it to the calibration mark with chromatographic-grade methanol to prepare a scutellarin reference solution with a concentration of 0.2 mg·mL^{-1}. Precisely transfer appropriate volumes of this standard solution into eight 10 mL volumetric flasks and dilute each to volume to obtain scutellarin solutions with concentrations of 1 mg·mL^{-1}, 5 mg·mL^{-1}, 10 mg·mL^{-1}, 20 mg·mL^{-1}, 40 mg·mL^{-1}, 50 mg·mL^{-1}, 80 mg·mL^{-1}, and 100 mg·mL^{-1}, respectively. Inject each sample three times, with an injection volume of 20 μL per sample, to plot the standard curve for scutellarin.

2.3 Establishment of HPLC Analytical Method

2.3.1 Analysis of Results on UV Absorption Peaks:

UV spectral analysis was performed on three breviscapine solutions of varying concentrations, with the results depicted in Figure S1. It was observed that as the

concentration escalated, the absorbance values correspondingly increased. Notably, the most intense absorption peak for breviscapine was identified at 335 nm. Consequently, it is established that 335 nm serves as the optimal detection wavelength for breviscapine during chromatographic analysis.

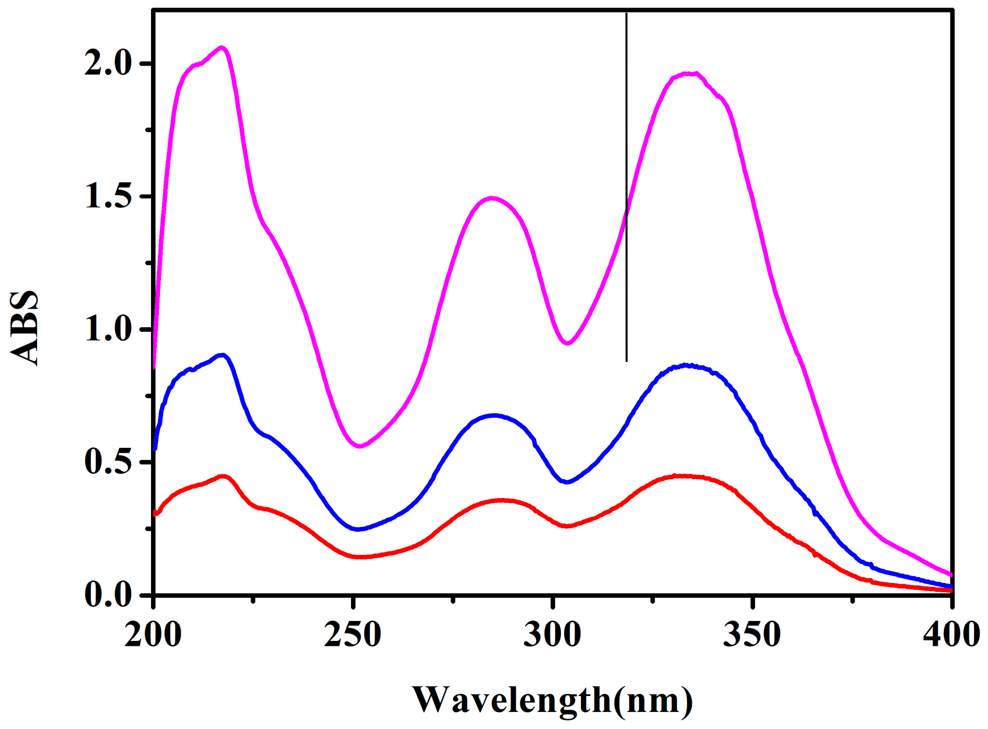

Figure S1 UV absorption spectrum of breviscapine solution

2.3.2 Chromatographic Conditions for the Determination of Breviscapine Content:

The chromatographic parameters employed for quantifying breviscapine are specified as follows: a C18 column (250 mm × 4.6 mm, 5 μm particle size); detection wavelength set at 335 nm; mobile phase composed of water and acetonitrile in a ratio of 80:20; column temperature maintained at 30°C; flow rate adjusted to 1 mL/min; injection volume fixed at 20 μL; retention time recorded as 9.954 min; and theoretical plate number ensuring a minimum of 2500.

2.3.3 Standard curve

The standard curve depicted in Figure S2 illustrates the calibration curve for breviscapine. UV analysis has confirmed that breviscapine exhibits a peak absorption at 335 nm. By analyzing breviscapine solutions of varying concentrations under specific chromatographic conditions, a standard curve was constructed using concentration as the abscissa and peak area as the ordinate. Furthermore, the linear regression equation derived from the calculations is: $A = 12.37C + 0.36$ ($R^2 = 0.9998$), indicating a strong linear correlation between peak area and concentration within the concentration range of 1 to 100 mg/mL.

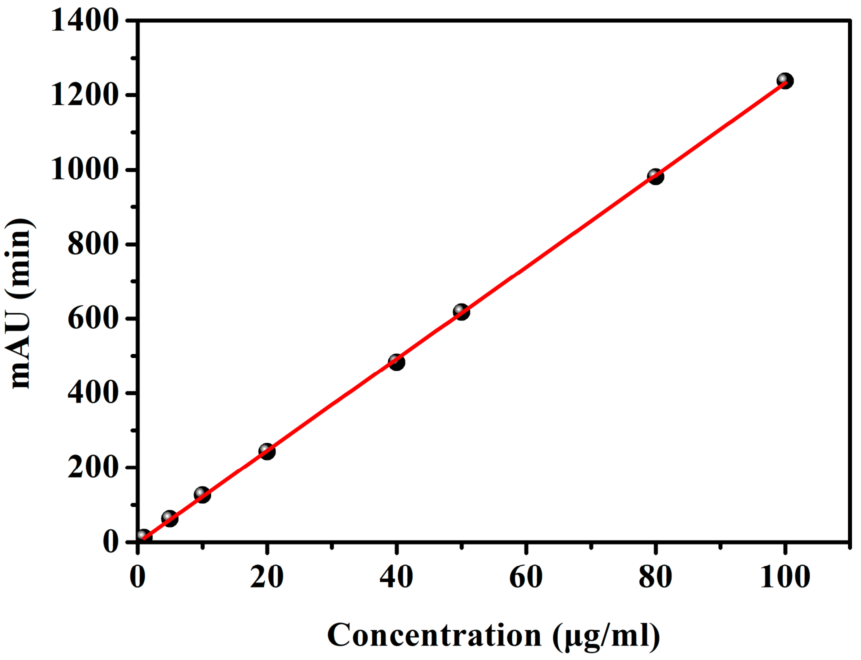

Figure S2 Breviscapine standard curve

2.4 Methodological Investigation

2.4.1 Analysis of Specificity Experimental Results

Figure S4 illustrates the peak profile of breviscapine at 335 nm, while Figure S5 displays the peak spectrum of the composite carrier material L57&PGP-PEG-PAMAM at the same wavelength. It is evident from the figures that, at a retention time of 9.954 min, a distinct peak is observed solely in the breviscapine solution, whereas no peak is detected in the L57&PGP-PEG-PAMAM solution at this retention time. This observation suggests that the composite material does not introduce any interference during the experimental detection of breviscapine.

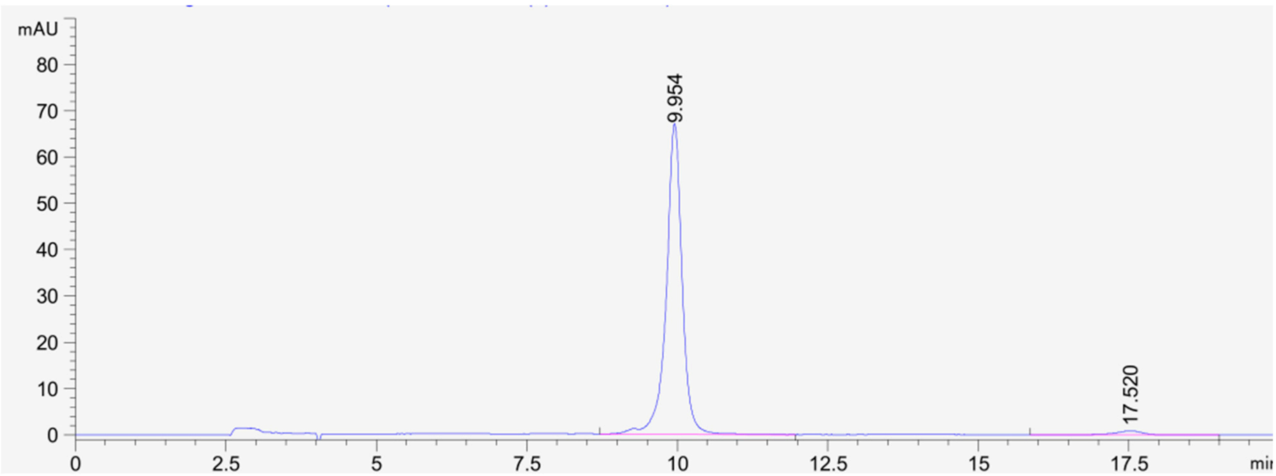

Figure S3 Chromatogram of breviscapine

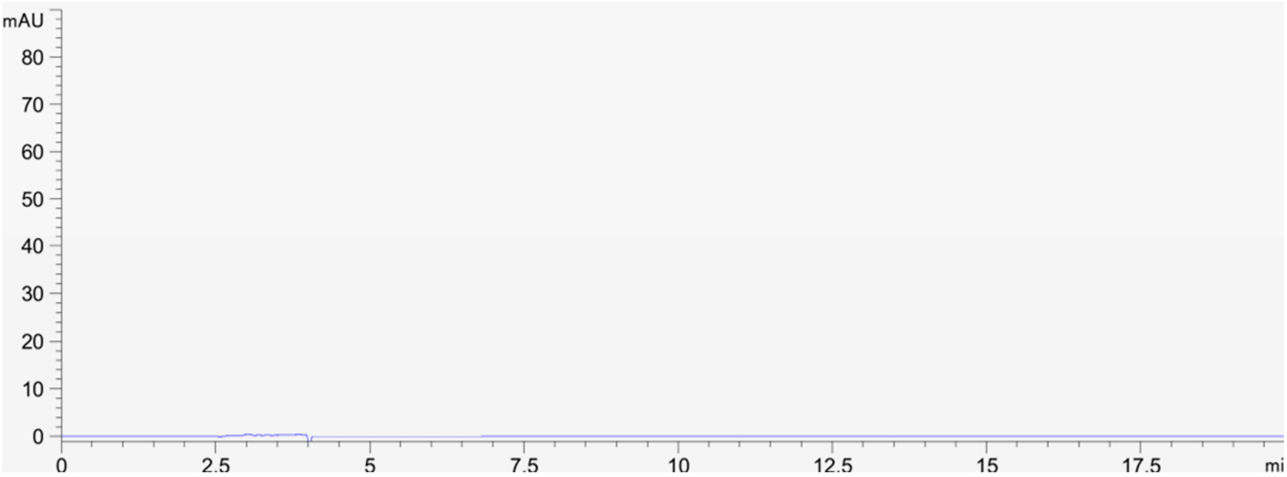

Figure S4 Chromatogram of L57&PGP-PEG-PAMAM

2.4.2 Precision Test

In accordance with the aforementioned methodology, prepare a breviscapine solution at a concentration of 5 mg·mL^{-1}. Subsequently, perform sample injection analysis under the predetermined chromatographic conditions. Determine the peak area and compute the RSD value. The results are shown in Table S5.

Table S5 Peak area, concentration and RSD value of breviscapine measured six times

Serial number	Peak area (mAU*min)	Measured concentration (μg/mL)
1	62.399	5.015
2	62.555	5.028
3	62.639	5.035
4	61.946	4.979
5	62.498	5.023
6	62.588	5.031
Average value	62.438	5.018
RSD	0.408%	

2.4.3 Recovery Rate Test

Three sets of sample solutions were prepared, containing sample amounts of 20 μg, 100 μg, and 300 μg, respectively. Equal quantities of breviscapine were added to each solution, followed by analysis using the aforementioned HPLC detection method. Each sample was injected three times to calculate the recovery rate of the spiked sample and the RSD value. The results are presented in Table S6. Based on the calculated results, the recovery rate complies with the requirements, indicating relatively accurate outcomes.

Table S6 Method recovery of breviscapine

Sample	Sample content (μg)	Addition (μg)	Measured value (μg)	percent recovery (%)	Average recovery rate (%)	RSD (%)
1	20	51.26	71.48	101.12		
2	20	51.26	71.23	99.85	100.31	0.61
3	20	51.26	71.25	99.95		
4	100	51.26	152.23	100.97		
5	100	51.26	149.98	98.72	99.81	1.13
6	100	51.26	150.98	99.72		
7	300	51.26	351.65	100.13	100.09	0.38

Sample	Sample content (μg)	Addition (μg)	Measured value (μg)	percent recovery (%)	Average recovery rate (%)	RSD (%)
8	300	51.26	350.66	99.8		
9	300	51.26	352.25	100.33		

3.Pharmacodynamic Study

3.1 Materials and Apparatus

3.1.1Experimental Instruments and Equipment

Table S7 The main instrument, model and manufacturer of the experiment

InstrumentName	Model	Manufacturer
Film Spreader	PPTHK-21B	Leike Company
Upright microscope	ECLIPSE Ni	NIKON Corporation
Microplate Reader	Anthos2010	Zhengzhou Bosai Bioengineering Co., Ltd
Ultra-Thin Semiautomatic Microtome	SQ2125	Leike Company
Microscopic Image Analysis System	DS-Ri2	NIKON Corporation
pipette	P2、P10、P20、P100、P200、P1000	Gilson P-type Pipette Company
Adjustable high-speed homogenizer	FSH-2	Nanjing Jiaozi Teng Scientific Equipment Co., Ltd

3.1.2Experimental Reagents

Table S8 Experimental reagents, purity and factory

Reagent name	Specification	manufacturer
chloral hydrate	Analytical Reagent	Qingdao Yulong Seaweed Co., Ltd
Chloro-2,3,5-trichlorophenyl tetrazolium (TTC) reagent kit	Batch number 20190407	Nanjing Jiancheng Bioengineering Research Institute
SD rats	200 g~220 g	Shanghai Slack Laboratory Animal Co., Ltd
Lamplighter extract	Analytical Reagent	Nanjing Chunqiu Bioengineering Co., Ltd
IL-15 cytokine	ELISA kit	R&D Systems (Minneapolis, USA)
IL-27 cytokine	ELISA kit	R&D Systems (Minneapolis, USA)

3.2 Establishment of rat cerebral ischemia-reperfusion injury model

SD rats were kept fasting for 12 hours before surgery. After being injected with chloral hydrate anesthesia, a partial incision was made at the midline of the neck to expose the bilateral carotid arteries. The external carotid artery was ligated at a position as far as possible from the bifurcation of the common carotid artery. A slipknot was tied around the internal carotid artery and the left common carotid artery. Finally, a notch was cut at a position about 3 mm away from the common carotid artery, and a 0.28 mm nylon thread was inserted into the internal carotid artery along this notch to a depth of about 1.9 cm, hindering blood flow to the brain. Two hours after surgery, the nylon thread was removed to restore blood flow in the cerebral

arteries of the rats, completing the ischemia-reperfusion process. The sham operation group did not undergo ligation and nylon thread treatment, and the other steps were the same.

3.3 Neurobehavioral function scoring

Neurological function scoring was performed 24 hours after ischemia-reperfusion. The higher the score, the more severe the injury. A score of 0 indicates normal activity of all four limbs, 1 indicates flexion of the forelimbs without free extension, 2 indicates involuntary rotation to the right side when walking, 3 indicates severe lateral circling behavior during walking, and 4 indicates no spontaneous activity and consciousness impairment. The behavioral states are shown in Figure S6: A represents flexion and inability to extend the contralateral forelimbs, B represents severe circling or toppling to the contralateral side, and C represents no spontaneous activity accompanied by consciousness impairment.

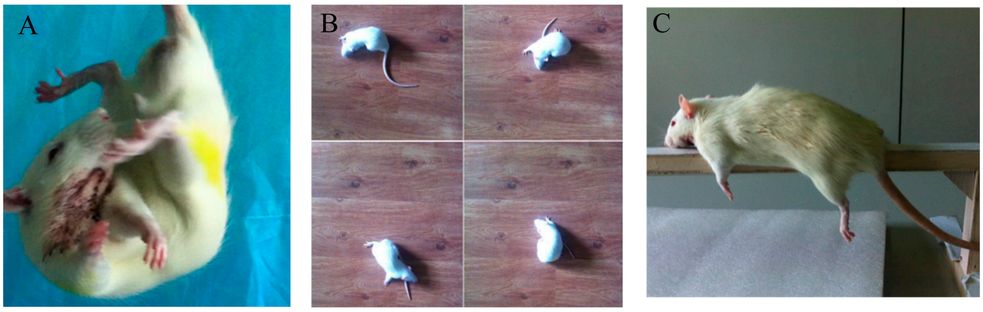

Figure S5 Nerve function scoring criteria

3.4 Measurement of cerebral infarction area

The cerebral infarction area was detected using the TTC staining method. After cerebral ischemia-reperfusion surgery, the rats were euthanized by decapitation, and the brains were removed intactly. The brain tissue was sliced along the coronal plane at approximately 2 mm intervals, and the processed brain tissue was placed in TTC staining solution. It was then incubated at 37℃, fixed with formaldehyde, and finally photographed.

3.7 Pathological examination of cerebral ischemic stroke by HE staining

After sacrificing the rats, dissect them and remove their intact brains. Place the brains in 4% formaldehyde fixative for 12 hours, then perform alcohol dehydration and other treatments. Cut the brains into sections with a thickness of approximately 4 μm, and finally perform HE staining. Seal the slides with neutral gum, and observe the pathological tissue changes in the rats before and after drug administration post-surgery.

3.8 Measurement of inflammatory cytokines IL-15 and IL-27 in brain tissue

3.8.1 Sample preparation

After cerebral ischemia-reperfusion surgery in rats, observe for 24 hours, then remove the brain tissue under low temperature and blot the blood on the tissue with

filter paper. Accurately weigh the wet weight of the brain tissue with a balance, add an appropriate amount of physiological saline to the right brain tissue at low temperature, and homogenize it with an adjustable high-speed homogenizer. Centrifuge the homogenate, transfer the supernatant to a beaker, and store it in the freezer.

3.8.2 Measurement of inflammatory cytokines IL-15 and IL-27 levels

The contents of inflammatory cytokines IL-15 and IL-27 in brain homogenate were detected using the enzyme-linked immunosorbent assay (ELISA) double antibody sandwich method to investigate the anti-inflammatory effect of the L57&PGP-PEG-PAMAM nanomedicine carrier loaded with breviscapine.

3.8.3 Data statistics

The experimental data obtained were analyzed for statistical significance using SPSS 19.0. All data were expressed as mean ± standard deviation ($x \pm s$). One-way ANOVA was used to compare and analyze multiple groups of data. $P < 0.01$ and $P < 0.05$ indicate statistically significant data.
